# Actin organization and endocytic trafficking are controlled by a network linking NIMA-related kinases to the CDC-42-SID-3/ACK1 pathway

**DOI:** 10.1371/journal.pgen.1007313

**Published:** 2018-04-02

**Authors:** Vladimir Lažetić, Braveen B. Joseph, Sarina M. Bernazzani, David S. Fay

**Affiliations:** Department of Molecular Biology, College of Agriculture and Natural Resources, University of Wyoming, Laramie, WY; NYU School of Medicine, UNITED STATES

## Abstract

Molting is an essential process in the nematode *Caenorhabditis elegans* during which the epidermal apical extracellular matrix, termed the cuticle, is detached and replaced at each larval stage. The conserved NIMA-related kinases NEKL-2/NEK8/NEK9 and NEKL-3/NEK6/NEK7, together with their ankyrin repeat partners, MLT-2/ANKS6, MLT-3/ANKS3, and MLT-4/INVS, are essential for normal molting. In *nekl* and *mlt* mutants, the old larval cuticle fails to be completely shed, leading to entrapment and growth arrest. To better understand the molecular and cellular functions of NEKLs during molting, we isolated genetic suppressors of *nekl* molting-defective mutants. Using two independent approaches, we identified CDC-42, a conserved Rho-family GTPase, and its effector protein kinase, SID-3/ACK1. Notably, CDC42 and ACK1 regulate actin dynamics in mammals, and actin reorganization within the worm epidermis has been proposed to be important for the molting process. Inhibition of NEKL–MLT activities led to strong defects in the distribution of actin and failure to form molting-specific apical actin bundles. Importantly, this phenotype was reverted following *cdc-42* or *sid-3* inhibition. In addition, repression of CDC-42 or SID-3 also suppressed *nekl*-associated defects in trafficking, a process that requires actin assembly and disassembly. Expression analyses indicated that components of the NEKL–MLT network colocalize with both actin and CDC-42 in specific regions of the epidermis. Moreover, NEKL–MLT components were required for the normal subcellular localization of CDC-42 in the epidermis as well as wild-type levels of CDC-42 activation. Taken together, our findings indicate that the NEKL–MLT network regulates actin through CDC-42 and its effector SID-3. Interestingly, we also observed that downregulation of CDC-42 in a wild-type background leads to molting defects, suggesting that there is a fine balance between NEKL–MLT and CDC-42–SID-3 activities in the epidermis.

## Author summary

Protein kinases are key molecular regulators that act by modifying the structures and activities of proteins within the cell. Members of the NEK family of protein kinases regulate cell division and the formation of specialized organelles called cilia. Accordingly, mutations in the human NEK genes have been implicated in a number of diseases including cancer and maladies that result from defective cilia. To better understand the biological functions of NEK kinases, we have undertaken studies in the model system nematode, *Caenorhabditis elegans*. In this study, we found that NEK kinases regulate the organization of actin, a major component of the cytoskeleton and a player in many cellular processes. In the absence of normal NEK kinase activity, actin fails to form proper filamentous structures during worm development, which leads to growth arrest. Interestingly, these defects in *nekl* mutants can be partially reversed by simultaneously reducing the activities of CDC42 or ACK1, two conserved proteins that are key regulators of actin dynamics. Other findings suggest that NEK kinases control actin organization through functional interactions with CDC42 and ACK1. These studies provide a new link between NEK kinases and the actin cytoskeleton and, together with other reports, suggest that these functions may be conserved in humans.

## Introduction

Members of the NIMA-related kinase (NEK) family are conserved serine/threonine kinases found in fungi, plants, and animals. The original member of the family, Never in Mitosis A (NIMA), was discovered in the filamentous fungus *Aspergillus nidulans*, where it promotes cell cycle progression [1, 2]. More recent analysis has indicated that NIMA interacts with components of the Endosomal Sorting Complex Required for Transport (ESCRT) to control normal polarized growth [3]. Most mammalian genomes encode eleven NEKs, termed NEK1–NEK11 [4], which have a number of distinct functions including roles in cell cycle progression, ciliogenesis, DNA damage response, and inflammasome activation [5–14]. Correspondingly, a number of NEKs have been implicated in human diseases including cancer, polycystic kidney disease, *situs inversus*, cardiopathies, paucity of bile duct syndrome, and Majewski syndrome [5, 15–24].

The genome of the nematode *Caenorhabditis elegans* encodes four NEK-like proteins, termed NEKL-1–NEKL-4 [25]. NEKL-3 is a close ortholog of mammalian NEK6 and NEK7, whereas NEKL-2 is most similar to NEK8 and NEK9 [25, 26]. We have previously shown that NEKL-2 and NEKL-3, together with three conserved ankyrin repeat partners, MLT-2/ANKS6, MLT-3/ANKS3, and MLT-4/INVS, are required for the completion of molting [25–27]. During molting, larvae release their old apical extracellular matrix (ECM), termed the cuticle, and synthesize a new one underneath, thereby allowing for further growth and development [28]. Whereas null mutations in *nekls* or *mlts* lead to a complete failure to shed the old cuticle during the first larval molt, hypomorphic alleles typically arrest during the second molt and exhibit a partial release of the old cuticle [25–27]. Our previous studies have suggested that the role of NEKL–MLT proteins in molting may be linked to their regulation of endocytic trafficking, which is critical for the normal molting process [25–27]. Whether the perturbation of normal trafficking is primarily responsible for molting defects in *nekl* and *mlt* mutations, however, is currently unresolved.

In this study, we further clarify the mechanism through which the NEKL–MLT pathway is involved in molting. More specifically, by using two independent screens, we have linked the NEKL–MLT pathway with the Rho-like GTPase, CDC-42/CDC42, and its conserved effector protein, SID-3/ACK1. CDC42 was initially identified as an essential cell polarity and budding regulator in the yeast *Saccharomyces cerevisiae* and its activity is in large part controlled through its association with GTP (in the active state) and GDP (in the inactive state) [29, 30]. Studies in mammals have shown that CDC42 becomes activated in response to different intracellular and extracellular signals and, depending on the type of the stimulus, can interact with a wide variety of effectors to control diverse cellular functions. These include actin reorganization and polarization, intracellular trafficking, polarized growth, microtubule polarization, and septin organization [31–41]. CDC42 is thus considered to be a key regulator in the establishment and maintenance of cellular polarity, a function that is essential for normal proliferation, differentiation, and morphogenesis [34]. ACK1 is a non-receptor tyrosine kinase and serine/threonine protein kinase that is implicated in cell morphology, migration, survival, growth, and proliferation [42–44]. More specifically, ACK1 functions in trafficking and clathrin-mediated endocytosis through binding to the epidermal growth factor receptor and clathrin [45–47], and also regulates the formation of actin filaments by phosphorylating both the Wiskott-Aldrich syndrome protein (WASP) and cortactin [48, 49]. In addition, ACK1 has been reported to promote the activation state of CDC42 [50]. In *C*. *elegans*, mutations in *sid-3* affect the transfer of dsRNA between cells, suggesting a possible connection to endocytosis and RNA import [51]. Nevertheless, the functions of SID-3 are not well characterized in *C*. *elegans*, and previous studies have not linked SID-3 functions to CDC-42 in this species.

Our study for the first time functionally connects CDC-42/CDC-42 and SID-3/ACK1 with the NIMA-related kinase network. Moreover, we demonstrate that these pathways are critical for the regulation of actin-dependent ECM remodeling and vesicular trafficking in the polarized epithelium of *C*. *elegans*.

## Results

### Inhibition of CDC-42 suppresses molting defects in *nekl-3* mutants

**Fig 1:**
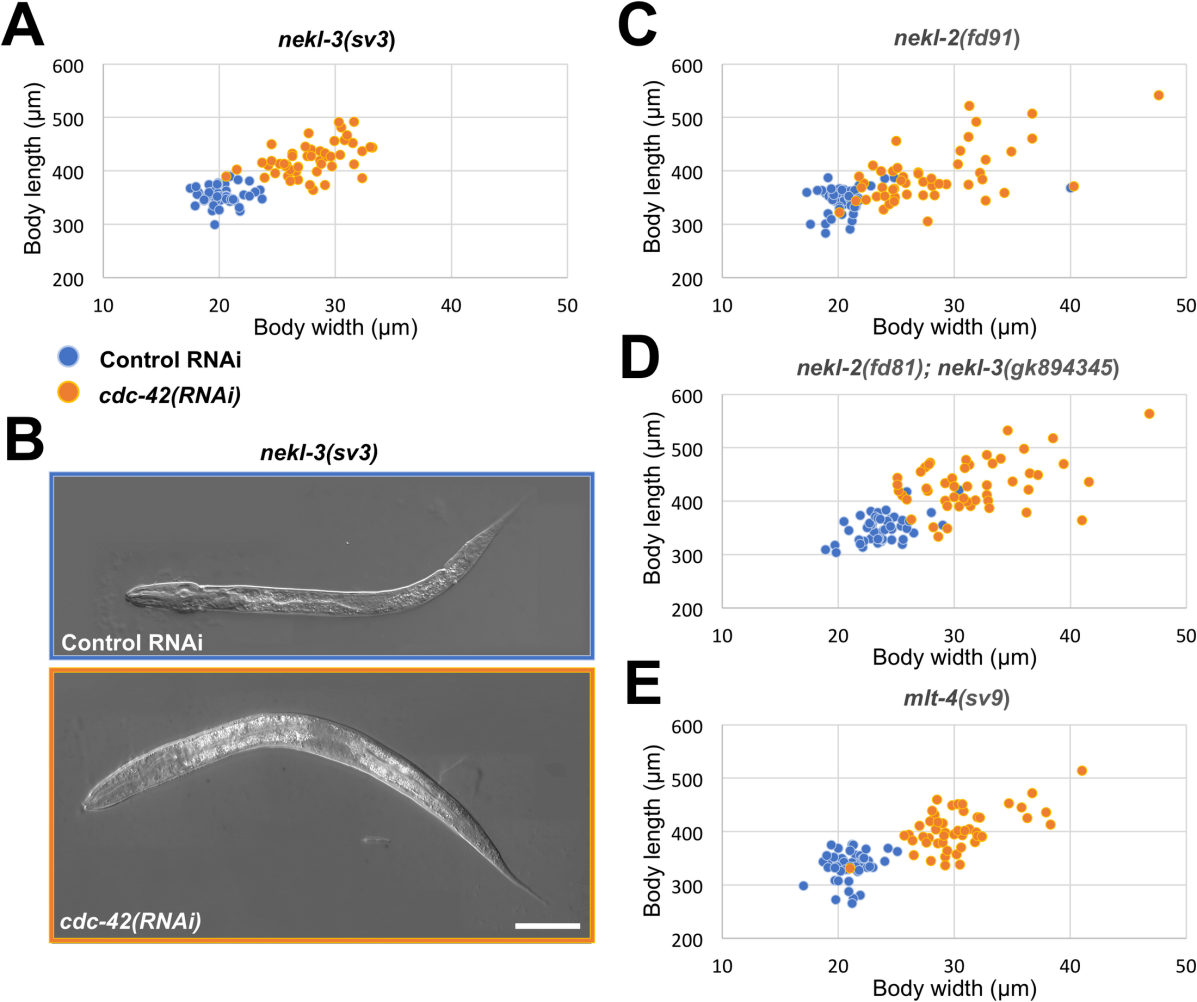
*cdc-42(RNAi)* suppresses molting defects in *nekl–mlt* hypomorphic mutants. (A,B) Graphic representation of body dimensions (A) and representative DIC images (B) of *nekl-3(sv3)* animals grown on control (blue) and *cdc-42(RNAi)* (orange) plates. Bar size in B = 50 μm. (C–E) Graphic representation of body dimensions of *nekl-2(fd91)* (C), *nekl-2(fd81); nekl-3(gk894345)* (D), and *mlt-4(sv9)* (E) animals grown on control RNAi (blue dots) and *cdc-42(RNAi)* (orange dots). Each dot in graphs in A and C–E represent one animal (n = 50 for each genotype and RNAi treatment). Animals of all examined genetic backgrounds increase in size when grown on *cdc-42(RNAi)* as compared with animals grown on control RNAi plates.

We have previously shown that mutations in components of the NEKL–MLT network lead to a spectrum of molting defects during larval development. In the case of *nekl-3(sv3)* hypomorphic mutants, larvae typically arrest at the second (L2/L3) molt and exhibit a stereotypical corset phenotype, whereby the unshed L2 cuticle constricts a significant portion of the central body region [25–27]. To gain insights into the underlying mechanisms controlling molting, as well as targets and components of the NEKL–MLT network, we carried out a screen for RNAi suppressors of *nekl-3(sv3)* molting defects. Among the genes identified, an RNAi clone targeting *cdc-42* led to penetrant suppression of *nekl-3(sv3)*, such that most treated animals progressed through L3 and L4 stages as compared with control animals that arrested at the L2/L3 transition (Fig 1A and 1B). We also tested the ability of *cdc-42(RNAi)* feeding to suppress defects in *nekl-3(gk506)* null mutants, which arrest during the L1/L2 molt and exhibit complete encasement within both the L1 and L2 cuticles [25]. In contrast to the *nekl-3(sv3)* hypomorphic allele, suppression of *nekl-3(gk506)* by *cdc-42(RNAi)* was relatively weak but did lead to an increase in the average width of treated animals (S1A Fig).

### Repression of CDC-42 suppresses defects in multiple *nekl–mlt* mutant backgrounds

To test if a reduction in CDC-42 can suppress loss-of-function phenotypes in other members of the NEKL–MLT network, we examined the effects of *cdc-42(RNAi)* feeding on the hypomorphic alleles of *nekl-2*(*fd91*) and *mlt-4*(*sv9*). In addition, we tested *cdc-42(RNAi)* on *nekl-2(fd81); nekl-3(gk894345)* double mutants. Notably, single mutants of *nekl-2(fd81)* and *nekl-3(gk894345)* are aphenotypic, whereas ~ 99% of double mutants arrest as larvae with molting defects [26]. On control RNAi plates, all three strains displayed near uniform L2/L3 arrest (Fig 1C–1E), consistent with previous reports [25, 26]. RNAi feeding of *cdc-42* led to partial suppression of molting defects in all three strains, whereby animals developed through L3 and L4 stages and in some cases progressed to adulthood (Fig 1C–1E). Suppressed adult animals, however, appeared to be sterile, which is likely due to a requirement for *cdc-42* in the germline [52, 53]. In addition, we tested for *cdc-42(RNAi)* suppression of the *nekl-2(gk839)* null allele, which leads to arrest with a complete encasement phenotype at the L1/L2 molt [25–27]. Similar to findings for *nekl-3(gk506)*, *cdc-42(RNAi)* led to a significant increase in larval width (S1B Fig), although suppression was quite weak. The inability of *cdc-42(RNAi)* to robustly suppress null alleles of *nekl-2* and *nekl-3* indicates that reduction of *cdc-42* cannot fully compensate for the complete loss of NEKL activity.

Because hypomorphic alleles are not available for the two additional characterized members of the *nekl–mlt* network, *mlt-2* and *mlt-3*, we used a combined RNAi-feeding approach to assay for *cdc-42* suppression. Animals were fed mixtures of bacteria expressing RNAi against either *mlt-2* or *mlt-3*, together with either *cdc-42* or control RNAi. Although suppression under these conditions was weaker than that observed for the hypomorphic alleles described above, *cdc-42(RNAi)* led to a significant increase in body width (S2 Fig), which may be due to partial release of the unshed cuticle. Taken together, these results demonstrate that depletion of CDC-42 can alleviate molting defects in all tested members of the conserved *nekl–mlt* network.

### CDC-42 function is required for molting in *C*. *elegans*

Given our observation that partial loss of *cdc-42* activity can lead to suppression of molting defects in *nekl–mlt* mutants, we were interested to determine if perturbation of CDC-42 levels might have an effect on molting in an otherwise wild-type background. Injection of *cdc-42* dsRNA, which induces a strong reduction in CDC-42 activity in the germline, leads to severe defects in the early embryonic development of F1 progeny because of disruptions in cell polarity and spindle orientation [52, 54]. In contrast, *cdc-42*(*gk388*) null homozygous mutants derived from heterozygous mothers complete embryogenesis but arrest later in development, likely because maternal CDC-42 provided by the germline is sufficient for progression through early stages of development [52–55].

**Fig 2:**
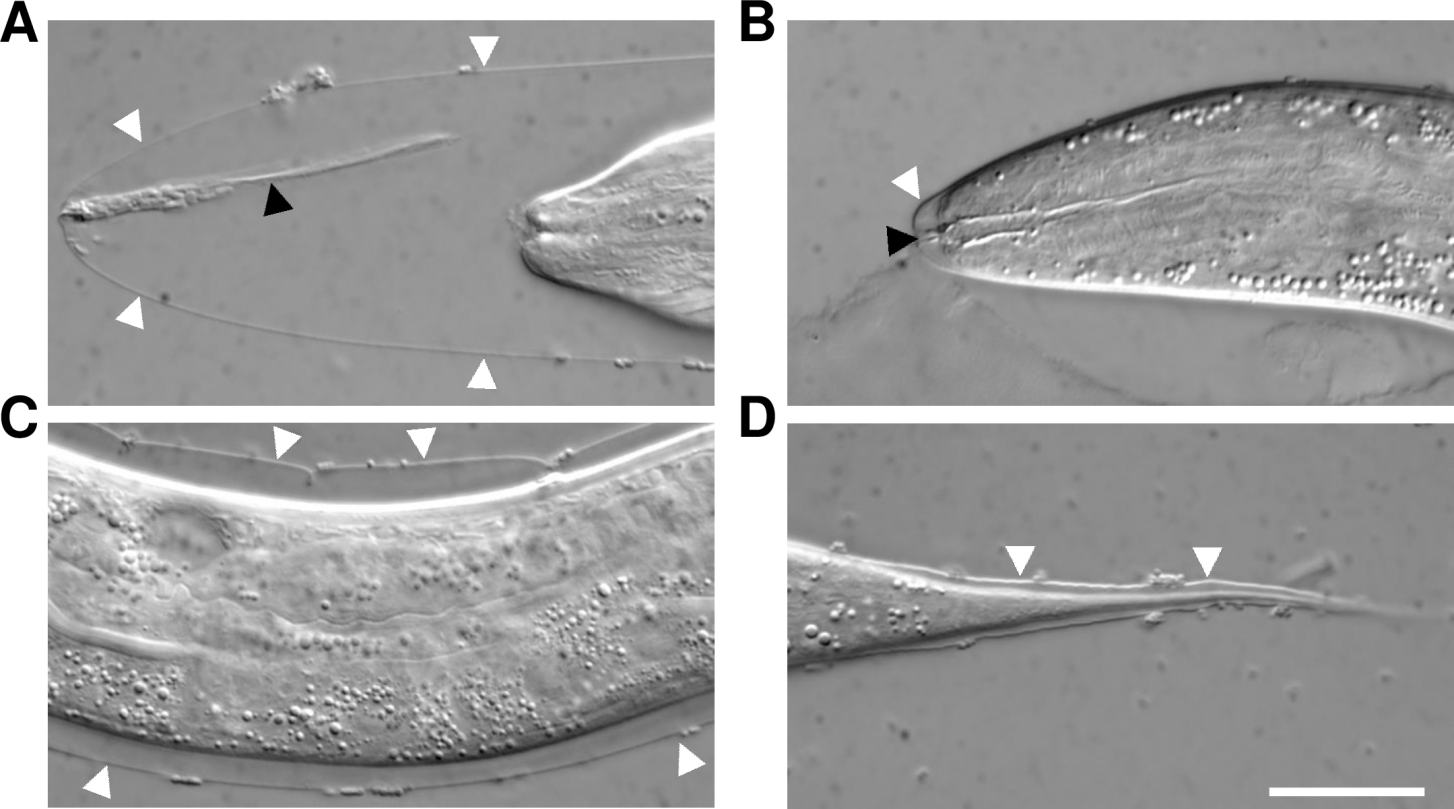
Molting defects in *cdc-42* null mutants. (A–D) Molting-defective phenotypes of *cdc-42(gk388)* homozygotes in the head region (A,B), mid-body region (C), and tail region (D). Note that *cdc-42(gk388)* homozygotes are generated by heterozygous mothers, which provide maternal CDC-42 required for embryogenesis. Arrowheads indicate detached old external (white) and buccal (black) cuticles. Bar size in D = 20 μm for A–D.

Interestingly, we found that 32% (n = 79) of *cdc-42(gk388)* homozygous progeny derived from heterozygous mothers displayed clear molting defects. Most commonly, cuticle detachments were observed in the head and tail regions, but in some cases shedding defects were visible in other locations (Fig 2). Based on P-cell divisions, the majority of *cdc-42* homozygotes arrested at the L3 stage, although 14% of examined animals progressed to L4 or adult stages (n = 79). Injection of genomic DNA encoding wild-type *cdc-42* into a balanced *cdc-42(gk388)* strain resulted in normal molting in the F1 generation of transformed larvae (n>20), demonstrating that the molting defects in this strain are specifically attributable to a lack of zygotic *cdc-42* expression. Taken together, our findings indicate that whereas weak inhibition of CDC-42 can partially compensate for a reduction in *nekl–mlt* activities, CDC-42 is nevertheless required for normal molting.

### CDC-42 is expressed in the epidermis and colocalizes with NEKL–MLT components

**Fig 3:**
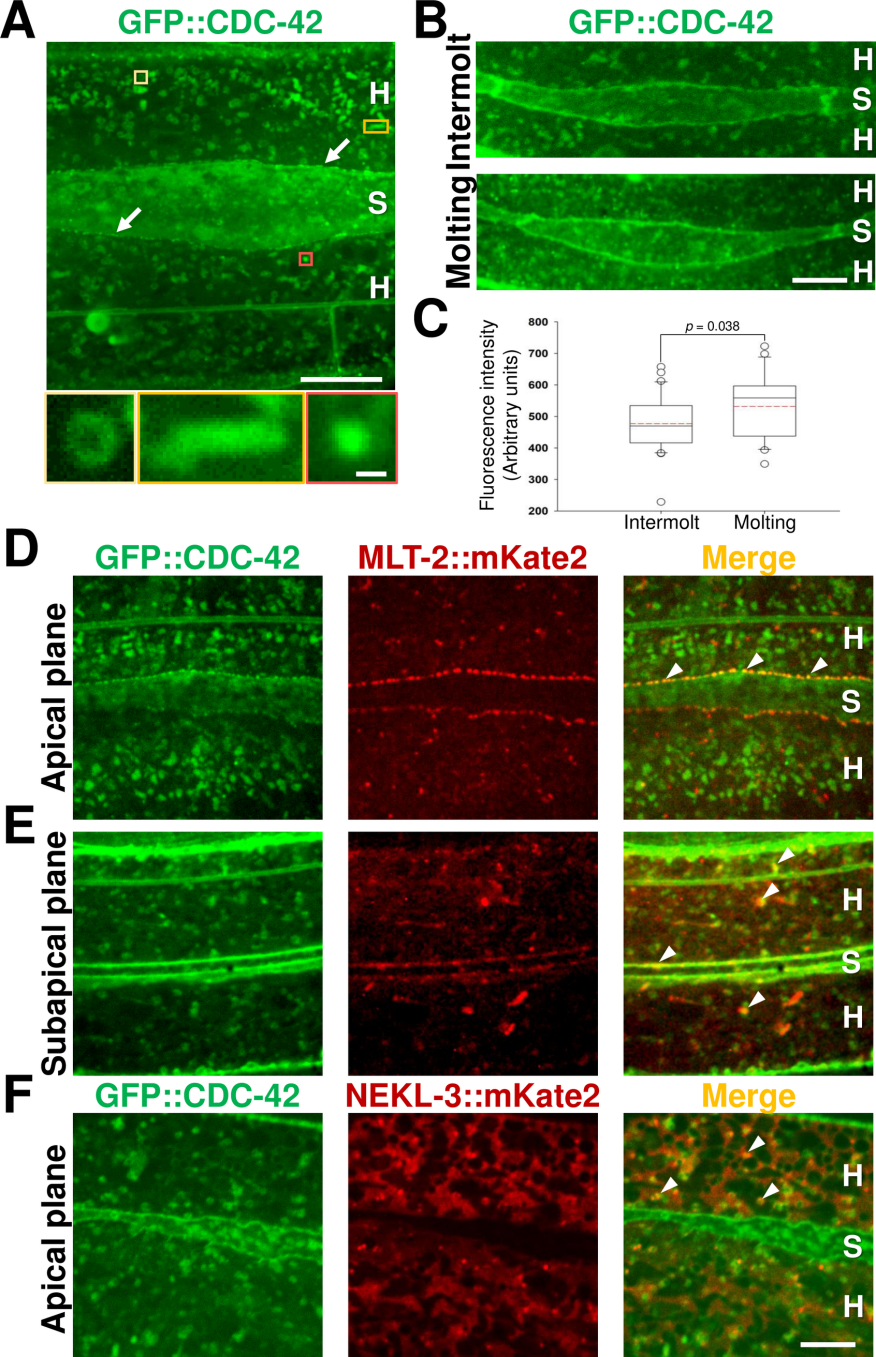
GFP::CDC-42 expression and colocalization with NEKL–MLT components. (A) GFP::CDC-42 expression pattern in the apical epidermis of an adult wild-type animal. Colored boxes indicate regions that are magnified in lower three images to show the different structures associated with GFP::CDC-42. Arrows indicate GFP::CDC-42 accumulation at the boundary between hyp7 and the seam cell. (B) GFP::CDC-42 expression is similar in intermolt (L2 intermolt) and molting (L2/L3) wild-type larvae. (C) Box-and-whisker plot of GFP::CDC-42 expression level measurements in intermolt and molting wild-type larvae. The box spans 50% of data that are closest to the median value (black line). Mean values for each data set are represented by dotted red lines. Whiskers indicate ranges of non-outlier values. Outliers that differ by ≥1.5-fold from the lower or upper quartiles are marked with white dots. (D–F) Colocalization analysis of GFP::CDC-42 with MLT-2::mKate2 and NEKL-3::mKate2 in L4 larvae. GFP::CDC-42 colocalizes with MLT-2::mKate2 at the boundary region between hyp7 and seam cells (D,E), as well as with subapical puncta in other regions of hyp7 (E). (F) GFP::CDC-42 and NEKL-3::mKate2 partially colocalize in regions of hyp7 that are not adjacent to the seam cell boundary. White arrowheads indicate representative regions showing marker colocalization. H, hyp7; S, seam cell. Bar sizes in A (upper panel) = 10 μm, A (lower panel) = 0.5 μm, B = 5 μm, F = 5 μm in D–F.

Because the epidermis is the primary tissue that modulates molting in *C*. *elegans* [25–27, 56], we next examined the pattern of CDC-42 expression in the larval epidermis. A functional GFP::CDC-42 reporter (S1 Table) was expressed in epidermal syncytia (hyp1–hyp11) as well as in lateral specialized epithelial seam cells (Fig 3A). GFP::CDC-42 accumulated in rounded, rod-like, or irregularly shaped structures throughout the cytosol that were enriched in the apical region of hyp7. GFP::CDC-42 was also observed in ring-shaped structures, suggesting that CDC-42 is associated with the membranes of vesicles (Fig 3A). Furthermore, smaller CDC-42 puncta accumulated at the boundary between the main epidermal syncytium hyp7 and seam cells (Fig 3A). We failed to observe any obvious differences in the pattern of GFP::CDC-42 expression in molting versus intermolt animals (Fig 3B), suggesting that CDC-42 localization may not be grossly altered during the molting program. We did, however, detect a statistically significant increase in mean levels of GFP::CDC-42 expression in molting versus intermolt animals, although these changes were relatively modest (Fig 3C).

We next asked if CDC-42 colocalized with components of the NEKL–MLT network that we previously examined using functional CRISRP-generated reporters (S1 Table) [25, 26]. In apical regions of hyp7, extensive colocalization between MLT-2::mKate2 and GFP::CDC-42 puncta was observed in 100% of animals (n = 33), primarily at the boundary of seam cells (Fig 3D and S3A Fig), although colocalization was also observed in more medial regions of hyp7 (Fig 3E and S3B Fig). GFP::CDC-42 also colocalized with a subset of bright NEKL-3::mKate2 puncta in 8/8 animals. Specifically, co-localization was observed in the apical portion of hyp7 in regions non-adjacent to the seam cell. (Fig 3F and S3C Fig). Given that we previously described two independent complexes containing NEKL-2–MLT-2–MLT-4 and NEKL-3–MLT-3 [26], our results indicate that CDC-42 partially colocalizes with both complexes during development.

### The NEKL–MLT network controls CDC-42 localization in the epidermis

**Fig 4:**
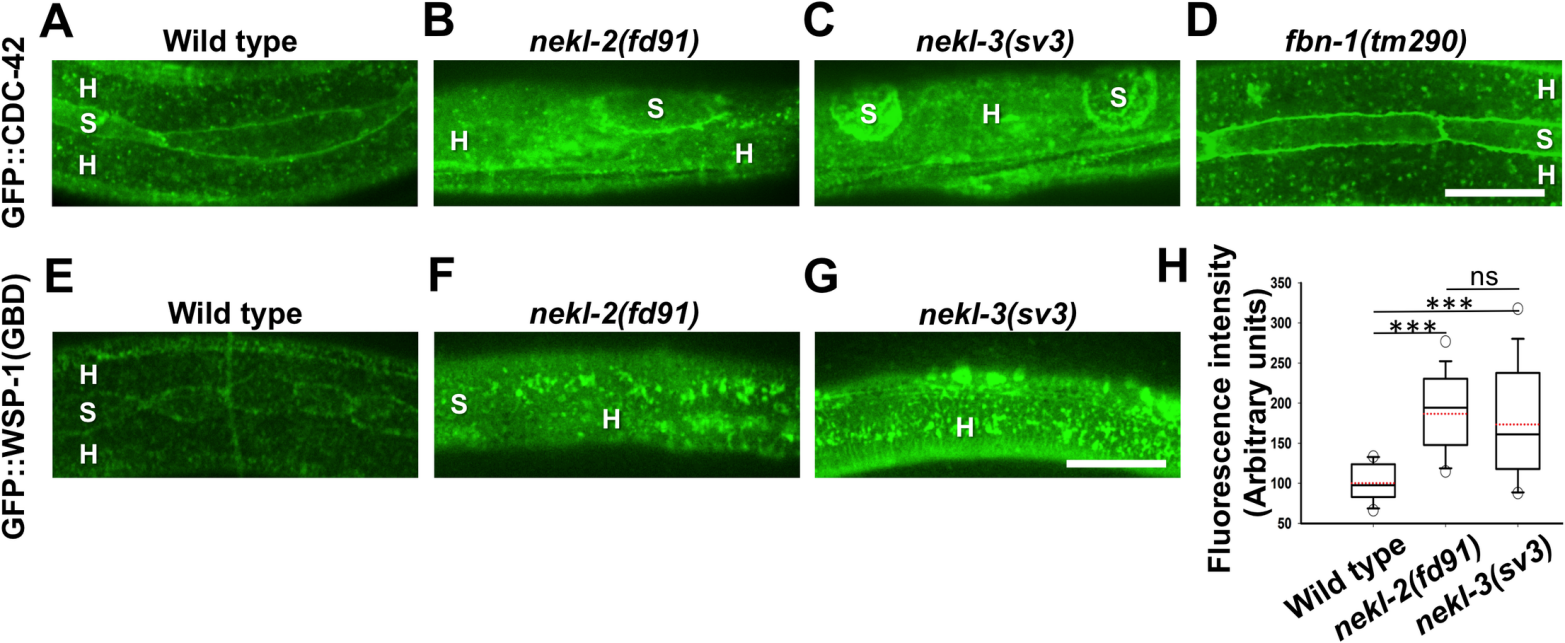
NEKL-2 and NEKL-3 control CDC-42 localization and activity. (A–D) Normal GFP::CDC-42 expression in the apical epidermal plane during the second molt (A) is altered in *nekl-2(fd91)* (B) and *nekl-3(sv3)* (C) mutants but is unaltered in *fbn-1(tm290)* molting-defective controls (D). (E-H) Wild type expression of a GST::GFP::WSP-1(GBD) reporter for active CDC-42 (E) is changed and higher in *nekl-2(fd91)* (F) and *nekl-3(sv3)* (G) mutants. Bar sizes in D and G = 10 μm in A–G. Change in GST::GFP::WSP-1(GBD) intensity is shown in graph in H. A Student's t-test was used to analyze differences in expression. Three asterisks represent significant differences in intensity (p ≤ 0.001), ns–not significant.

Given the observed functional and spatial connections between CDC-42 and NEKL–MLT components, we next determined if the localization of CDC-42 is dependent on the NEKL–MLT network. Notably, GFP::CDC-42 epidermal localization was altered in 89% (n = 53) of *nekl-2(fd91)* and 79% (n = 53) of *nekl-3(sv3)* molting-defective larvae (Fig 4A–4C). In particular, apical expression of CDC-42::GFP was altered either throughout the entire epidermis or within limited regions. Changes included smearing (diffuse expression) of the GFP::CDC-42 marker as well as increased aggregation. Although GFP::CDC-42 was highly enriched at seam cell boundaries in *nekl-2(fd91)* and *nekl-3(sv3)* mutants, consistent with wild type, seam cells in these mutants were abnormally small and round relative to wild-type controls (Fig 4A–4C), suggesting a non-autonomous role for NEKL-2 and NEKL-3 in seam cell processes. In addition, we observed severe defects in GFP::CDC-42 localization in 62% (n = 21) of *mlt-3(RNAi)* animals, including smearing and aggregation of the marker within the apical epidermis (S4A and S4B Fig). These findings are consistent with a role for the NEKL–MLT network in directly or indirectly regulating epidermal CDC-42 subcellular localization.

To address the possibility that the observed changes in GFP::CDC-42 localization in *nekl* and *mlt* mutants were due to indirect effects caused by improper shedding of the cuticle, we examined GFP::CDC-42 expression in several unrelated molting-defective backgrounds depleted for FBN-1, a fibrillin-like protein (Fig 4D); MLT-11, a putative protease inhibitor (S4C Fig); or QUA-1, a hedgehog-like signaling molecule (S4D Fig). We observed that the pattern of GFP::CDC-42 localization and seam cell morphology appeared normal in 92% (n = 26) of *fbn-1(tm290)* mutants, as well as 71% (n = 14) of *mlt-11(RNAi)*, and 100% (n = 10) of *qua-1(RNAi)* worms, indicating a specific role of the NEKL–MLT protein network in the control of CDC-42 localization.

### CDC-42 activity is altered in *nekl-2* and *nekl-3* mutants

In addition to gross changes in CDC-42 expression, we were interested to determine if CDC-42 activation was affected by loss of *nekl* function. As a dynamic switch, CDC-42 cycles between GTP-bound active and GDP-bound inactive forms. To assay CDC-42 activity, we made use of two independent reporters of CDC-42 activation. We first examined a GST::GFP::WSP-1(GBD) reporter that associates with GTP-bound active CDC-42 and is expressed under the control of the *cdc-42* promoter [57]. In the case of wild type larvae we observed a punctate pattern of expression near the apical surface, which was very similar to our observations for the GFP::CDC-42 reporter (Fig 4E). Notably, we observed both qualitative and quantitative differences in patterns of GST::GFP::WSP-1(GBD) in wild-type larvae and *nekl-2(fd91)* and *nekl-3(sv3)* single mutants. GST::GFP::WSP-1(GBD) tended to form aggregations in the mutants (Fig 4F and 4G). In addition, apical expression levels of GST::GFP::WSP-1(GBD) in mutants were nearly twice the levels observed in wild type larvae (Fig 4H), suggesting increased CDC-42 activity in the mutants.

To confirm these results, we used another reporter for CDC-42 activity, WSP-1(CRIB)::mCherry, which is expressed exclusively in the epidermis under the control of the *lin-26* promoter [58]. In wild-type larvae, WSP-1(CRIB)::mCherry was primarily observed in small puncta and filament-like structures throughout the epidermis of molting and intermolt animals, with occasional localization within slightly larger aggregates (S5A Fig). The different expression pattern of this reporter in comparison to both GFP reporters for active and total CDC-42 may be due to difference in the fluorescence tag and its C terminal localization. Although we observed some variability in the pattern of WSP-1(CRIB)::mCherry between individual larvae, no consistent differences were detected between molting (n = 25) and intermolt (n = 31) animals. In contrast, we observed a strong increase in the size (S5 Fig) of WSP-1(CRIB)::mCherry puncta in 93% of *nekl-2(fd91)* (n = 28) and 96% of *nekl-3(sv3)* (n = 28) larvae, as well as an increase in the number of puncta in some animals. This included the formation of very large accumulations that were never observed in wild type (S5 Fig). In contrast to *nekl* mutants, we observed a wild-type pattern for WSP-1(CRIB)::mCherry in 91% of *fbn-1(tm290)* molting-defective mutants (n = 21) (S5D Fig), and the size of mCherry puncta in *fbn-1(290)* mutants was much closer to that of wild type (S5 Fig), suggesting that the observed effects on CDC-42 activity are likely specific to perturbation of the NEKL–MLT network. Collectively, these findings indicate that NEKL–MLT components affect both CDC-42 subcellular localization as well as its activity levels.

We also tested the effect of expressing two predicted CDC-42 hyperactive variants (G12V and Q61L) [54, 59–61] in the epidermis using the *dpy-7* promoter, which drives specific expression within epidermal cells [62]. Whereas expression of wild-type CDC-42 from transgenic arrays was well tolerated and gave rise to transmitting lines, both the G12V and Q61L CDC-42 variants were highly toxic, leading to severe morphological defects and accompanying embryonic and L1 larval lethality in most GFP-marked F1 progeny (n>1000). In one representative experiment using *cdc-42*(G12V) with the co-injection marker *sur-5*::*GFP*, we observed 57% embryonic lethality and 27% early L1 lethality (n = 165) of GFP-positive worms whereas *cdc-42*(Q61L) led to 55% embryonic lethality and 22% L1 lethality (n = 176). This level of lethality was observed using a range of DNA concentrations and is likely due to the deleterious effects of hyperactive CDC-42 on the epidermis of embryos undergoing morphogenesis. Approximately 20% of F1s did not arrest as embryos or L1 larvae but failed to produce stable transmitting lines. F1s that escaped early arrest were typically highly mosaic for expression of the GFP-containing array with a majority displaying some form of morphogenetic defect including molting deficits (S6 Fig). In some cases, we observed complete entrapment within the old cuticle at the L2/L3 stage, whereas other worms failed to shed only a portion of the cuticle or became entrapped at later stages (S6 Fig). These findings are consistent with our above data and indicate that epidermal-expressed hyperactive CDC-42 can cause defects in molting. One caveat to these studies, however, is that the observed effects on molting could be indirectly due to defects in epidermal cells induced by the CDC-42 gain-of-function mutants earlier in development. Nevertheless, molting phenotypes were observed in some animals that did not display obvious morphological defects (S6 Fig), suggesting that these effects are separable.

### Mutations in the *C*. *elegans* activated Cdc42-associated kinase 1 ortholog, SID-3, suppress molting defects in *nekl-2* and *nekl-3* mutants

**Fig 5:**
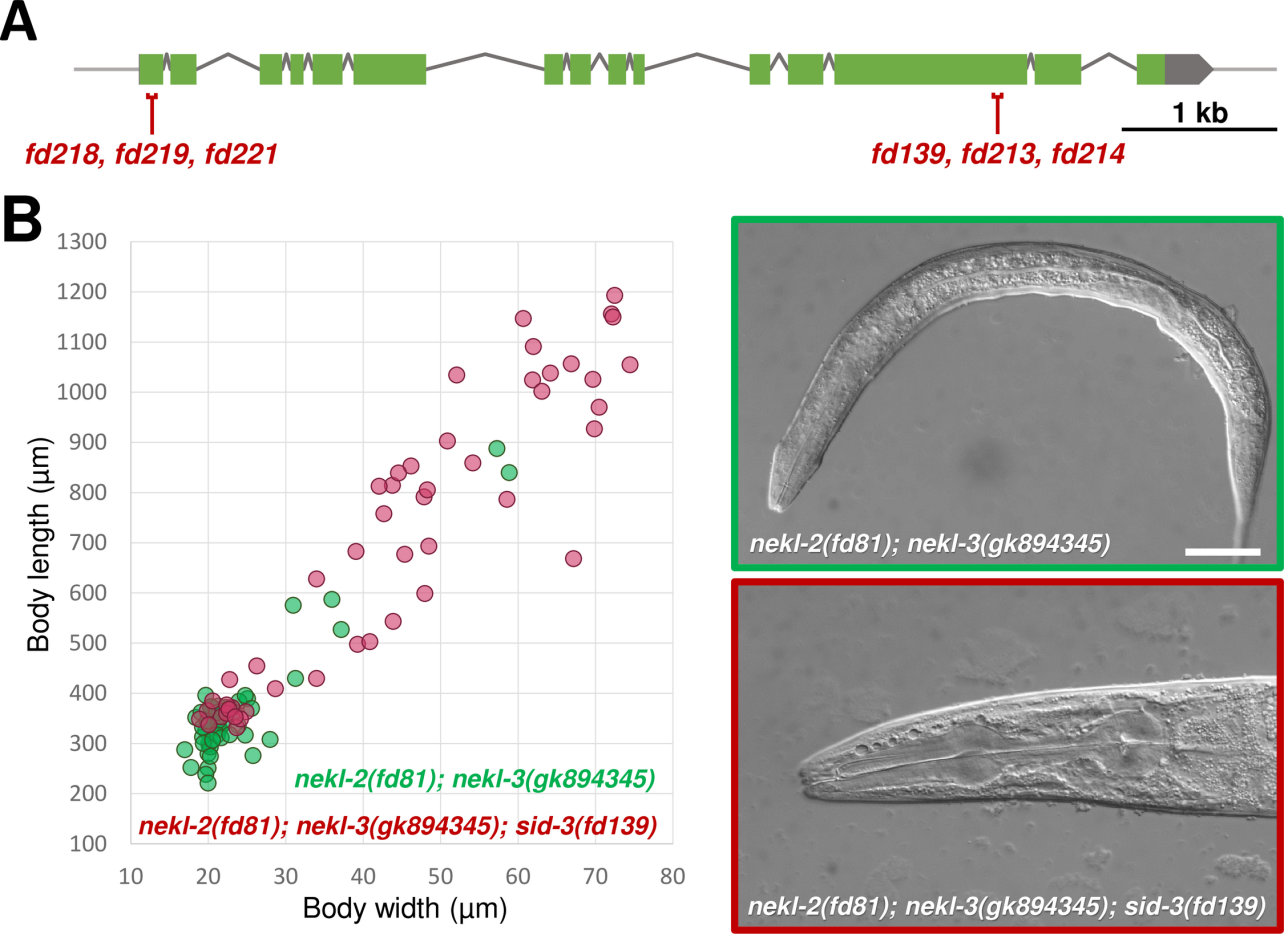
Mutations in *sid-3* suppress molting phenotypes of *nekl-2*; *nekl-3* hypomorphic mutants. (A) Schematic illustration of *sid-3* with indicated loss-of-function alleles (in red). (B) Comparative graphic representation of body dimensions and representative DIC images of *nekl-2(fd81); nekl-3(gk894345)* double mutants (green) and *nekl-2(fd81); nekl-3(gk894345); sid-3(fd139)* triple mutants (red). Each dot in the graph represents one animal (n = 50 for each genotype). Bar size in B = 30 μm.

In a parallel forward genetic screen [63], we identified a mutation, *fd139*, that strongly suppressed molting defects in *nekl-2(fd81); nekl-3(gk894345)* double mutants (Fig 5A and 5B). Molecular identification of *fd139* revealed it to affect *sid-3*, the ortholog of activated Cdc42-associated kinase 1 (ACK1). ACK1 was initially described as a positive regulator of CDC42 activity, although later studies have shown that it also functions as a CDC42 effector and has roles in actin regulation and intracellular trafficking [45–50]. In *C*. *elegans*, SID-3 regulates systemic RNAi and is expressed in the epidermis in a pattern that is similar to what we observed for CDC-42 [51].

*fd139* is a 5-bp deletion in *sid-3* exon 13, leading to a frameshift following Ser 989 of the SID-3 protein (B0302.1a). Using CRISPR/Cas9 methods, we generated five additional alleles of *sid-3*, all of which induced strong suppression of the corset phenotype in *nekl-2(fd81); nekl-3(gk894345)* animals (Fig 5A and S2 Table). This included two C-terminal (*fd213* and *fd214*) and three N-terminal (*fd218*, *fd219*, *fd221*) alleles, of which the latter group caused frameshifts within 60 bp downstream of the start codon. Notably, none of these alleles has any obvious phenotype on their own, consistent with a previous report [51]. The ability of the N-terminal *sid-3* alleles to suppress *nekl-2(fd81); nekl-3(gk894345)* mutants indicates that complete inactivation of SID-3 can suppress molting defects in the double mutants. These results further strengthen the functional connection between NEKL–MLT components and CDC-42 and also suggest that SID-3 cooperates with CDC-42 in the molting network. The viability of null mutations in *sid-3*, however, indicates that unlike CDC-42, SID-3 is not required for normal molting [51].

### NEKL–MLT components are required for normal actin organization in the epidermis

In mammals, CDC42 and Ack1 have important roles in the regulation of the actin cytoskeleton. Importantly, epidermal actin is reorganized during molts to form circumferentially oriented arrays, which have been proposed, though not formally demonstrated, to be critical for molting [64]. The observation that inhibition of both *cdc-42* and *sid-3* can suppress defects in *nekl–mlt* mutants further suggested that the NEKL–MLT network may control actin localization during molting.

**Fig 6:**
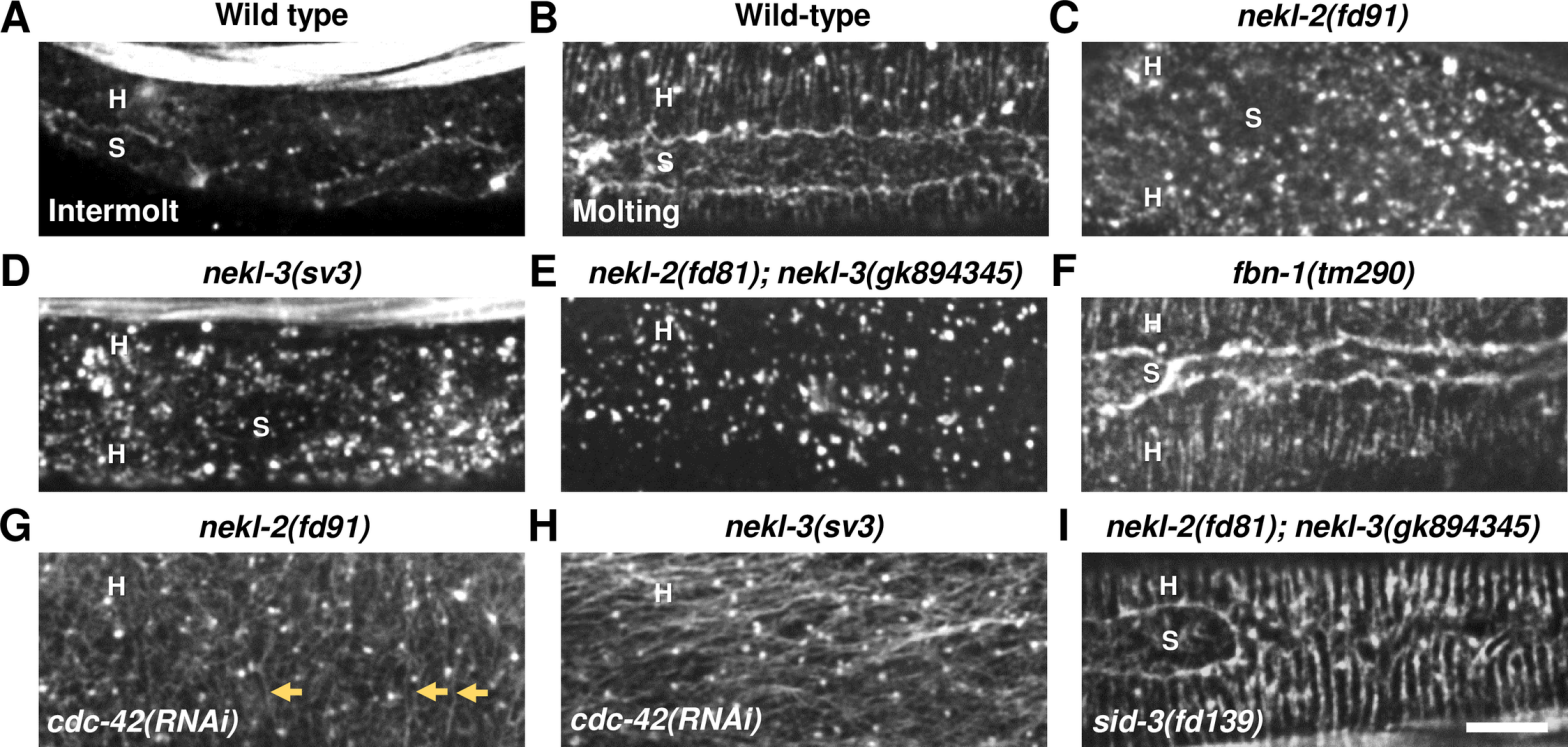
Actin organization in the epidermis is controlled by NEKL and CDC-42 pathway components. (A,B) Apical actin, which is largely diffuse in intermolt larvae (A), reorganizes into a network of parallel bundles during molting (B). The apical actin network is severely perturbed in *nekl-2(fd91)* (C), *nekl-3(sv3)* (D), and *nekl-2(fd81); nekl-3(gk894345)* (E) mutants, unlike in *fbn-1(tm290)* molting-defective control animals (F). (G,H) *cdc-42(RNAi)* partially restores normal actin organization in *nekl-2(fd91)* (G) and *nekl-3(sv3)* (H) mutants, including the formation of actin filaments (indicated by orange arrows). (I) *sid-3(fd139)* completely restores molting-specific apical actin organization in *nekl-2(fd81); nekl-3(gk894345)* double mutants. H, hyp7; S, seam cell. Bar size in I = 5 μm in A–I.

We first sought to recapitulate the observations of Costa et al. [64] using phalloidin staining of actin in fixed wild-type worms. We observed that intermolt L2 larvae generally showed very weak actin staining in the epidermis (Fig 6A), suggesting that actin in these animals may be largely diffuse. Occasionally, intermolt animals contained dispersed epidermal actin puncta as well as some accumulation at the seam cell boundary (Fig 6A). In contrast, phalloidin staining in molting animals was much stronger, and actin was clearly organized into parallel apical bundles, consistent with Costa et al. (Fig 6B) [64].

To test if epidermal actin organization is regulated by the NEKL–MLT network, we first examined actin staining in *nekl-2(fd91)* (n = 29) and *nekl-3(sv3)* (n = 18) hypomorphic animals. We failed to observe normal actin bundles in any of the examined animals from both genetic backgrounds. In most larvae, the pattern of actin differed dramatically from wild type in that puncta were more numerous and tended to form aggregations (Fig 6C and 6D). Moreover, although mutants occasionally contained short filamentous-like structures, they failed to complete the formation of parallel bundles observed in wild-type molting animals (S7 Fig). In 24% of *nekl-2(fd91)* and 11% of *nekl-3(sv3)* mutants we observed some actin filaments or rows of actin puncta in the dorsal and ventral areas of hyp7, usually above the body wall muscles (S7B and S7C Fig). Notably, patches with actin filaments were more frequently observed in body regions constricted by the old cuticle; it is possible that actin filaments formed and rapidly disassembled in the non-constricted regions. In addition, actin accumulations at the hyp7–seam-cell boundary were frequently undetectable.

Consistent with the above findings, actin was mislocalized in 100% (n = 38) of *nekl-2(fd81); nekl-3(gk894345)* double mutants, although to a lesser extent than in *nekl-2(fd91)* and *nekl-3(sv3)* single mutants (Fig 6E). Likewise, we observed that formation of filaments was initiated to varying extents in 68% (n = 38) of *nekl-2(fd81); nekl-3(gk894345)* mutants (S7D Fig) but failed to form complete rings. Similar to the single mutants, filaments were prevalent within, but not exclusive to, regions constricted by the cuticle. Large actin aggregates were observed in the posterior regions of *nekl-2(fd81); nekl-3(gk894345)* larvae, which are typically free of old cuticle. As a control, we also performed phalloidin staining with *fbn-1(tm290)* molting-defective animals and observed a normal molting-specific pattern of actin organization (n = 8) (Fig 6F), indicating that the effects observed for *nekl-2* and *nekl-3* depletion are not likely to be an indirect consequence of improper molting.

Collectively, our findings suggest that formation of actin bundles can be initiated but not completed in *nekl* hypomorphs. Furthermore, filament formation is more severely disrupted in stronger loss-of-function *nekl* mutants. The strong loss of function allele *nekl-3(sv3)* shows very subtle and rare formation of actin filaments, whereas actin bundle formation was more extensive when two weak loss of function alleles, *nekl-2(fd81)* and *nekl-3(gk894345)*, were combined. From these studies we conclude that NEKL-2 and NEKL-3 play an important role in actin organization during molting.

### Depletion of CDC-42 and SID-3 reverse actin organization defects in *nekl* mutants

Given that *cdc-42* or *sid-3* depletion can suppress molting defects in *nekl-2* and *nekl-3* mutants, we next determined if epidermal actin organization was restored in suppressed larvae. We observed that *cdc-42(RNAi)* had a strong effect on actin organization in both *nekl-2(fd91)* and *nekl-3(sv3)* backgrounds, both by reducing the occurrence of actin accumulations and by promoting the formation of actin filaments (Fig 6G and 6H). In the case of *nekl-2(fd91); cdc-42(RNAi)*, actin filaments were observed in 96% (n = 24) of animals, however, these filaments were frequently disorganized. 92% (n = 12) of *nekl-3(sv3); cdc-42(RNAi)* suppressed animals also contained numerous actin filaments, but these did not form parallel circumferential bundles and in some cases were in an anterioposterior orientation (Fig 6H). The observed differences between *nekl-2* and *nekl-3* strains may be due to differences in their specific allele strengths or because *cdc-42(RNAi)* can more efficiently suppress reduced NEKL-2 functions.

To further analyze effects of CDC-42 downregulation on actin organization in the apical epidermis, we carried out actin staining on *cdc-42(gk388)* molting defective mutants. Although many of these animals were mostly impermeable to phalloidin, we observed a range of abnormal actin patterns. Importantly, in 60% of mutants (n = 25) actin puncta were organized in parallel rows, reminiscent of molting-specific actin bundles in wild type (S8C and S8D Fig). Nevertheless, filaments connecting these puncta were often missing. Furthermore, the boundary with the seam cells in *cdc-42(gk388)* mutants was not always enriched in actin filaments. In the remainder of *cdc-42(gk388)* molting defective animals (40%), actin puncta and rare filaments showed abnormal actin organization patterns and did not form parallel rows (S8E and S8F Fig). Thus, loss of *cdc-42* activity in an otherwise wild-type background leads to alterations in the normal pattern of actin staining, although this phenotype was generally less severe than that observed in *nekl-2* and *nekl-3* mutants.

We also examined actin localization in suppressed and *nekl-2(fd81); nekl-3(gk894345) sid-3(fd139)* animals and observed either a normal molting-specific actin pattern in animals that contained filaments (n = 6) (Fig 6I) or a normal punctate intermolt actin pattern (n = 13). Taken together, our data imply that NEKL-2 and NEKL-3 influence the formation of epidermal apical actin structures during development and that this function is tightly balanced by the activities of CDC-42 and SID-3.

### Apical actin colocalizes with NEKL–MLT components

**Fig 7:**
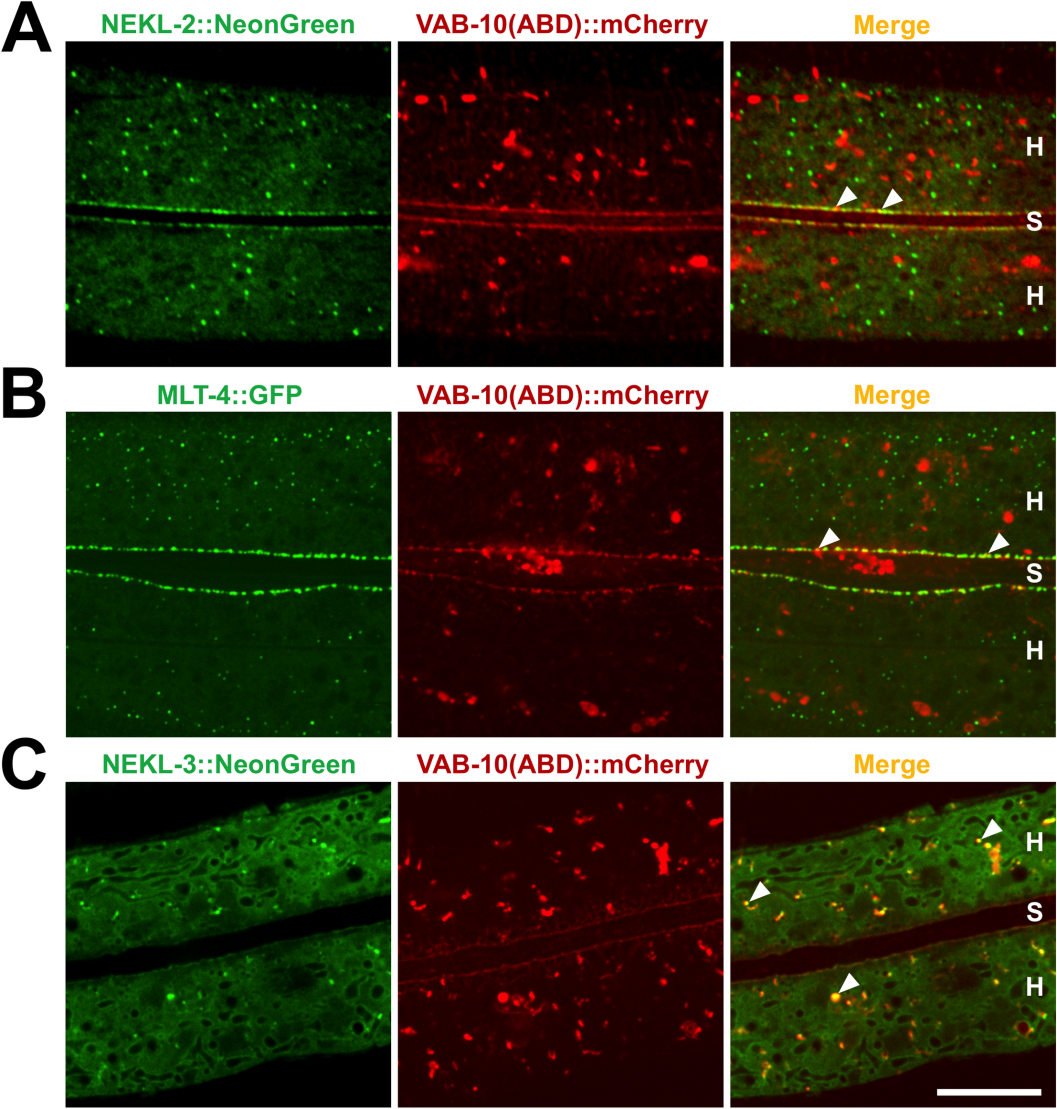
Colocalization of NEKL–MLT components with actin. (A,B) VAB-10(ABD)::mCherry, a marker for actin in live animals, colocalizes with NEKL-2::NeonGreen (A) and MLT-4::GFP (B) at the seam cell–hyp7 boundary. (C) NEKL-3::NeonGreen puncta extensively colocalize with VAB-10(ABD)::mCherry in other apical regions of hyp7. White arrowheads indicate representative regions of colocalization. H, hyp7; S, seam cell. Bar size in C = 10 μm in A–C.

Because NEKL–MLT components partially colocalize with CDC-42 and because *nekl* depletion led to a strong perturbation of the actin cytoskeleton, we next asked whether members of the NEKL–MLT network colocalize with actin. For these studies, we used a strain carrying an integrated *vab-10* actin-binding domain (ABD) mCherry fusion reporter, which can serve as a proxy for actin expression in live animals [65]. A CRISPR-generated reporter for NEKL-2::NeonGreen (S1 Table) strongly colocalized with the VAB-10(ABD)::mCherry reporter in 100% of worms (n = 15) at subapical regions of the epidermal–seam cell boundary, with little colocalization observed in other regions (Fig 7A and S9A Fig). Likewise, a CRISPR-generated reporter for MLT-4 (S1 Table), which forms a putative complex with NEKL-2 [26], showed a similar degree of overlap with VAB-10(ABD)::mCherry in the same region (n = 16) (Fig 7B and S9B Fig). Lastly, a CRISPR-generated reporter for NEKL-3 (S1 Table) was also found to strongly colocalize with VAB-10(ABD)::mCherry in hyp7 in 100% of examined animals (n = 11), however, in contrast to NEKL-2 and MLT-4, this overlap occurred throughout the cytoplasm and rarely at the seam cell boundary, where NEKL-3 is largely absent (Fig 7C and S9C Fig). Collectively, our data place members of the NEKL–MLT complex in close proximity to both actin and CDC-42, consistent with a role for NEKL–MLT proteins in regulating the actin cytoskeleton through the CDC-42–SID-3 pathway.

### Depletion of CDC-42 and SID-3 suppress trafficking defects in *nekl* mutants

**Fig 8:**
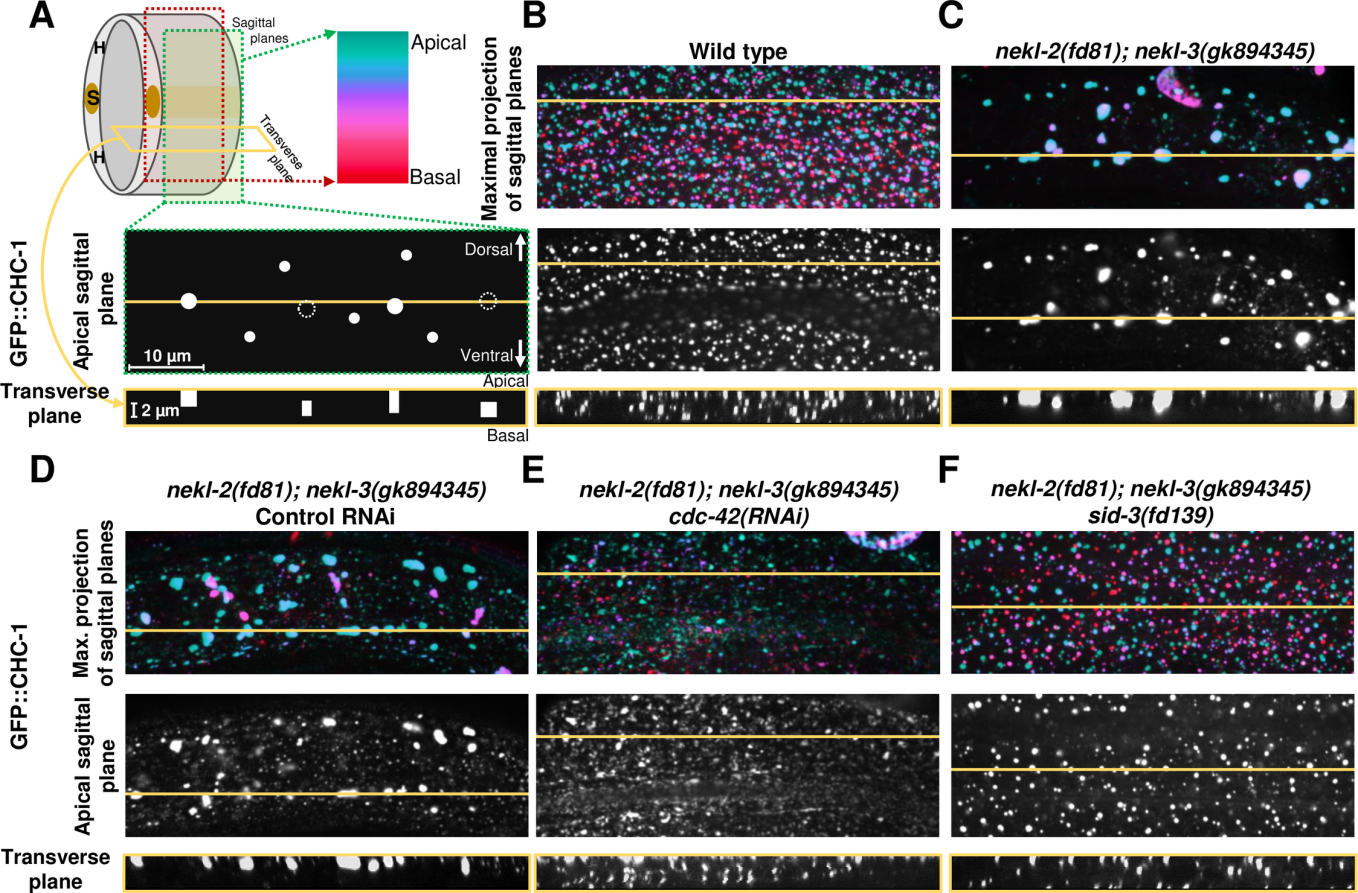
CDC-42 and SID-3 downregulation suppresses endocytosis defects in *nekl-2(fd81); nekl-3(gk894345)* double mutants. (A) Model depicting epidermal regions analyzed in this study. Expression of GFP::CHC-1 marker was examined in 24 sagittal planes through the epidermis. Color scale indicates the epidermal position of analyzed puncta in maximal projections of sagittal planes. Yellow line indicates location that was used to generate transverse plane projections from *z*-stacks of sagittal planes. In the apical sagittal plane illustration, filled white circles indicate apical puncta, whereas unfilled circles with dotted edges represent epidermal puncta that are present only in medial or basal planes. The transverse plane model indicates how selected puncta from the sagittal plane would appear along the apical–basal axis. (B) GFP::CHC-1 is expressed in small, well-distributed puncta throughout the epidermis in wild-type animals. (C,D) The GFP::CHC-1 expression pattern is severely altered in *nekl-2(fd81); nekl-3(gk894345)* double mutants grown on standard NGM (C) and control RNAi (D) plates, with the formation of large aggregates of the marker. (E,F) This phenotype can be partially reversed in *cdc-42(RNAi)*–treated animals (E) and in *nekl-2(fd81); nekl-3(gk894345); sid-3(fd139)* triple mutants (F). Scale bars and their sizes are indicated for corresponding sections in A.

We previously reported that inhibition of NEKL–MLT components leads to defects in endocytosis in the epidermis [25, 26]. Because orthologs of CDC-42 and SID-3 in other systems regulate several steps of endocytosis [40, 41, 45–47, 66, 67], we wanted to test if inhibition of CDC-42 and SID-3 could suppress trafficking defects in *nekl* mutants. To examine effects on trafficking, we used a marker for clathrin, GFP::CHC-1, which localizes to clathrin-coated pits at the apical membrane [25, 26] and was also distributed in a punctate pattern throughout the epidermis (Fig 8A and 8B). Consistent with our previous findings, 100% (n = 57) of *nekl-2(fd81); nekl-3(gk894345)* double mutants contained abnormal accumulations of the GFP::CHC-1 marker. Specifically, *nekl-2(fd81); nekl-3(gk894345)* mutants exhibited fewer total GFP::CHC-1 puncta but these puncta were larger (Fig 8C and 8D and S10 Fig) [26]. In some cases, GFP::CHC-1 accumulations were observed to extend from the apical hyp7 surface to more medial regions of the epidermis, whereas other accumulations were exclusively medial (Fig 8C and 8D). *cdc-42(RNAi)* was observed to fully restore normal GFP::CHC-1 morphology in small percentage (15%; n = 34) of suppressed *nekl-2(fd81); nekl-3(gk894345)* animals that reached adulthood (Fig 8E). In addition, we observed a slight increase in the number of GFP::CHC-1 puncta in *nekl-2(fd81); nekl-3(gk894345)* animals treated with *cdc-42(RNAi)* versus control, although these differences were not statistically significant (S10 Fig). In fact, abnormal clathrin aggregations were prevalent in the epidermis of many *cdc-42(RNAi)* animals that were partially or fully suppressed for molting defects (S11B Fig), suggesting that *cdc-42(RNAi)* may suppress molting defects through a mechanism that is at least partially independent of trafficking as judged by clathrin localization. In contrast, *sid-3(fd139)*, which induces stronger suppression of molting defects in *nekl-2(fd81); nekl-3(gk894345)* mutants than *cdc-42(RNAi)* (compare Fig 1D and Fig 5B), led to restoration of normal GFP::CHC-1 expression in 82% of animals (n = 39) (Fig 8F and S10 Fig). Very rarely, some aggregates could be observed in *nekl-2(fd81); nekl-3(gk894345); sid-3 (fd139)* triple mutants, although they were typically smaller than in non-suppressed double mutants (S11C Fig). In summary, our results indicate that inhibition of CDC-42 and its partner SID-3 can suppress molting phenotypes in mutants of the *nekl–mlt* network and can at least partially correct associated defects in actin patterning and trafficking.

## Discussion

We have shown that inhibition of the small Rho GTPase CDC-42 leads to the suppression of molting defects in *nekl–mlt* hypomorphic backgrounds. Likewise, we independently found that mutations in *sid-3*, the *C*. *elegans* ortholog of activated Cdc42-associated kinase 1, also suppress *nekl* mutants, indicating that CDC-42–SID-3 functions are closely connected to NEKL–MLT signaling in the epidermis. Furthermore, we have observed that *cdc-42* downregulation in wild type can lead to molting defects and developmental arrest, suggesting that there is a functional balance between NEKL–MLT and CDC-42 activities in the epidermis. This balance becomes particularly important during molting, when inhibition of either component impairs normal ECM remodeling. Moreover, our observation that inhibition of CDC-42 and SID-3 can suppress molting defects in *nekl–mlt* mutants indicates that NEKL kinases are most likely negative regulators of CDC-42–SID-3. This control could be at the level of CDC-42–SID-3 intrinsic activity, through effects on subcellular localization, or both.

**Fig 9:**
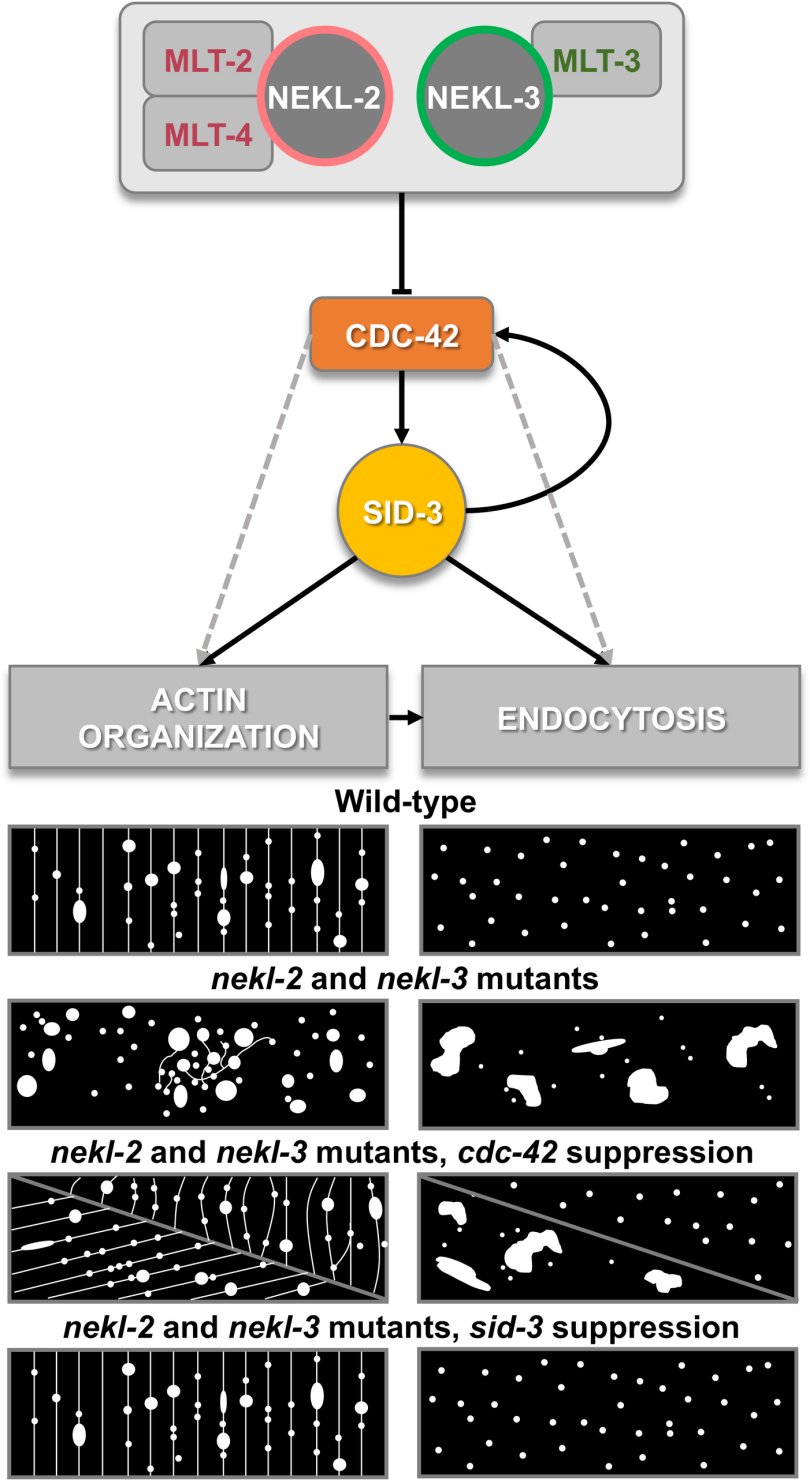
Summary model of observed results. NEKL-2 and NEKL-3 downregulation leads to aberrant actin and clathrin expression patterns in the epidermis, which can be partially suppressed by *cdc-42(RNAi)* or completely restored to the wild-type expression pattern by inactivating SID-3. For additional information, see Discussion.

Consistent with a strong functional link, we observed that CDC-42 partially colocalizes with components of both NEKL–MLT complexes, NEKL-2–MLT-2–MLT-4 and NEKL-3–MLT-3. Furthermore, normal GFP::CDC-42 localization and activity depend on NEKL-2 and NEKL-3, suggesting that there is both a spatial and functional interaction between CDC-42 and the NEKL–MLT protein network. Interestingly, independent proteomic studies to identify binding partners of mammalian NEK6 and NEK7 both identified CDC42 [68, 69]. These findings suggest that CDC42 interacts directly or indirectly with NEK6 and NEK7, the highly conserved mammalian orthologs of NEKL-3. Furthermore, a CDC42 modifier, Rho-GAP SNX26 (ARHGAP33), which is implicated in regulation of vesicular trafficking, has also been identified as interacting with NEK6 [68]. Because the phosphorylation targets of NEK kinases are largely unknown, we hypothesize that CDC42, its regulators, or its effectors could be controlled by NEK phosphorylation (Fig 9). This is consistent with findings that the phosphorylation status of CDC42 and its GEFs is important for their localization and activity [70–72].

CDC42 has important functions in the establishment of cell polarity, cytoskeletal reorganization and the regulation of endocytosis [34, 59, 73, 74]. In this study we analyzed the effects of *nekl–mlt* depletion on two CDC-42-dependent processes, actin remodeling and endocytosis, which are known to be important for molting in *C*. *elegans* [27, 64, 75]. It has been previously proposed that apical actin bundles reorganize at each molt to facilitate proper ECM formation [64]. In support of this model, we have shown that loss of NEKL-2 and NEKL-3 activity leads to defects in the pattern of apical actin, a failure to form molting-specific arrays of actin bundles, and defective molting (Fig 9). These apical actin bundles are proposed to contract as new cuticle is synthesized between the actin filaments, thus allowing for the formation of a new cuticle that is larger than the previous one [64]. Our finding that NEKL-2 and NEKL-3 depletion strongly perturb apical actin organization suggests a mechanism by which both detachment of the old cuticle and new cuticle synthesis might be impaired in *nekl–mlt* mutants. Namely, disorganization of the actin cytoskeleton may alter the distribution and timing of new cuticle synthesis and create an abnormal juxtaposition of the old and new cuticles, resulting in a failure to fully separate. Our observation that *nekl* mutants often contain incompletely formed molting-type actin bundles within constricted body regions supports this idea. In addition, colocalization data from this study indicate that components of the NEKL–MLT network and actin are partially coexpressed in the same subcellular compartments, which, together with CDC-42 colocalization data, further support a model for the specific control of actin organization by NEKL kinases through CDC-42. Consistent with this model, inhibition of CDC-42 and SID-3 can suppress actin localization defects in *nekl* mutants, suggesting that NEKL–MLT components are required to spatially and/or temporally repress CDC-42–SID-3 activity in the epidermis.

Interestingly, studies in mammalian cells have implicated MLT-4/INVS in apical actin regulation as INVS knockout mouse fibroblasts form excessive actin-rich filopodia during interphase as well as in mitosis [76]. Notably, filopodia formation is CDC42 dependent, and filopodia overproduction by mammalian cells is a characteristic of mutations that lead to higher CDC42 activity [77, 78]. Moreover, CDC42 activity increases in mammalian cells following depletion of INVS, consistent with INVS acting as a negative regulator of CDC42 [79]. Our study suggests that this function for MLT-4/INVS may be conserved in *C*. *elegans* and demonstrates for the first time that other components of the NEKL–MLT network control CDC42 activity and actin organization.

Both CDC42 and the actin cytoskeleton have been previously implicated in multiple aspects of vesicular trafficking. For example, actin may control the spatial organization of endocytic machinery components, promote the formation of membrane invaginations, create forces required for vesicle fission and internalization, and provide a barrier function that negatively regulates the rate of endocytosis [80–82]. Actin is essential for clathrin-mediated endocytosis in yeast, although its role in mammalian endocytosis may be more limited. Interestingly, actin is required for mammalian endocytosis at cell surfaces that are enriched for actin filaments [81]. Notably, such cortical enhancement of actin filaments is found at the apical surface of epidermal syncytium in *C*. *elegans* [64], providing a potential connection between actin and the regulation of vesicular trafficking during molting.

Importantly, endocytosis is a critical process for ECM remodeling in *C*. *elegans*, and inactivation of core endocytic components, including CHC-1, leads to molting defects [25–27, 75, 83–86]. One possible role of endocytosis in ECM remodeling may be in resorption and recycling of old cuticle components [27]. Furthermore, epidermal endocytosis has been implicated in the process of sterol uptake from the surrounding environment, and sterols have a proposed role in the synthesis of hormonal cues that initiate molting in nematodes [27, 56]. In addition, endocytosis is closely coupled to exocytosis [87], a process critical for cuticle synthesis [27]. Namely, the rate of exocytosis must be balanced with a commensurate level of endocytosis to allow for the recycling of membrane components back to intracellular compartments and to maintain a relatively constant cell surface area [87].

Previously, we have shown that the NEKL–MLT protein network regulates endocytosis in the epidermis [25, 26]. In this study, we found that highly penetrant trafficking defects in *nekl-2(fd81); nekl-3(gk894345)* double mutants can be partially or completely suppressed by CDC-42 and SID-3 downregulation. Nevertheless, we observed that suppression of molting defects was not always well correlated with the restoration of normal clathrin localization patterns. This could be due to different threshold requirements for the suppression of molting defects versus the normalization of clathrin-mediated endocytosis. For example, a partial correction of vesicular trafficking may be sufficient to prevent overt molting defects. Alternatively, impaired endocytosis may not be a primary cause of molting defects in *nekl–mlt* mutants, consistent with our earlier finding that trafficking alterations are not detected in all molting-defective animals depleted for components of the NEKL–MLT network [25, 26]. Instead, the observed endocytosis defects could be a secondary consequence of actin disorganization in *nekl–mlt* mutants, given that apical actin assembly and disassembly are critical for normal endocytosis [81, 82, 88–93]. In addition, it is possible that perturbation of both endocytosis and apical actin organization contribute to the observed molting defects in *nekl–mlt* mutants and that suppression can be achieved through compensatory effects on either process.

In summary, our data implicate the *C*. *elegans* orthologs of NIMA-related kinases NEK6, NEK7, NEK8, and NEK9, along with their conserved ankyrin-repeat binding partners, in the regulation of actin remodeling and intracellular trafficking through functional interactions with CDC42 and its effector ACK1. We propose that, by modulating CDC42–ACK1 activity, NEK kinases may control a wide variety of cellular processes including effects on actin morphology and endocytosis (our study), along with potential downstream effects on cell cycle–related processes and ciliogenesis. Future studies will be required to determine the generality of our observations along with the specific nature of this regulatory connection.

## Materials and methods

### Strains

*C*. *elegans* strains were maintained according to standard protocols [94] and were propagated at 21°C. Strains used in this study include FT1459 [*unc-119(ed3); xnIs506(cdc42p*::*gst*::*gfp*::*wsp-1[gbd]; unc-119[+])*], GR1395 [*mgIs49(mlt-10*::*gfp-pest)*], N2/Bristol (wild type), LH373 [*nekl-3(gk506); mnEx174(F19H6; pTG96)*], NL2099 [*rrf-3(pk1426)*], RT1378 [*pwIs528(gfp*::*chc-1)*], SP2734 [*mlt-4(sv9) V; mnEx173 (mlt-4(+); pTG96)*], SP2736 [*nekl-3(sv3)X; mnEx174(F19H6; pTG96)*], WS5018 [*cdc-42(gk388); opIs295 (cdc-42p*::*gfp*::*cdc-42; unc-119[+]) II*], WY1061 [*nekl-2(gk839); fdEx257(WRM0639aE11; WRM0636aD02; pTG96)*], WY1145 [*nekl-2(fd81); nekl-3(gk894345); fdEx286(pDF153[nekl-3(+)]; pTG96)*]; WY1165 [*nekl-2(fd91[Y84L*,*G87A]; fdEx278[pDF166 (nekl-2[+]; pTG96)])*], WY1217 [*nekl-2(fd81); nekl-3(gk894345); sid-3(fd139)*], WY1242 [*nekl-2(fd81); nekl-3(gk894345); pwIs528 (gfp*::*chc-1); fdEx257(WRM0639aE11; WRM0636aD02; pTG96)*], WY1331 [*nekl-2*::*NeonGreen*::*3xFlag (fd100); mcIs40(plin-26*::*vab-10(abd)*::*mCherry; myo-2*::*gfp)*], WY1332 [*nekl-3*::*NeonGreen*::*3xFlag (fd118); mcIs40(plin-26*::*vab-10(abd)*::*mCherry; myo-2*::*gfp)*], WY1333 [*mlt-4*::*gfp*::*3xFlag (fd114); mcIs40(plin-26*::*vab-10(abd)*::*mCherry; myo-2*::*gfp)*], WY1337 [*mlt-2*::*mKate2*::*3xFlag (fd95); cdc-42(gk388); opIs295 (cdc-42p*::*gfp*::*cdc-42; unc-119[+])II*], WY1338 [*nekl-3*::*mKate2*::*3xFlag (fd106); cdc-42(gk388); opIs295 (cdc-42p*::*gfp*::*cdc-42; unc-119[+])II*], WY1345 [*nekl-2(fd81); nekl-3(gk894345); sid-3(fd139); pwIs528(gfp*::*chc-1)*], WY1352 [*nekl-2(fd81); nekl-3(gk894345); sid-3(fd213)*], WY1353 [*nekl-2(fd81); nekl-3(gk894345); sid-3(fd214)*], WY1360 [*cdc-42(gk388); opIs295 (cdc-42p*::*gfp*::*cdc-42; unc-119[+])II; nekl-2(fd91); fdEx278 (pDF166 [nekl-2(+)]; pTG96)*], WY1361 [*cdc-42(gk388); opIs295 (cdc-42p*::*gfp*::*cdc-42; unc-119[+])II; nekl-3(sv3) X; mnEx174 (F19H6; pTG96)*], WY1374 [*sajIs31 (lin-26p*::*wsp-1[crib]*::*mCherry; unc-119R)*], WY1376 [*cdc-42(gk388); opIs295 (cdc-42p*::*gfp*::*cdc-42; unc-119[+])II; fbn-1(tm290); fdEx250 (sur-5*::*rfp*, *fbn-1 cDNA pMH281)*], WY1377 [*nekl-2(fd81); nekl-3(gk894345); sid-3(fd218)*], WY1378 [*nekl-2(fd81); nekl-3(gk894345); sid-3(fd219)*], WY1380 [*nekl-2(fd81); nekl-3(gk894345); sid-3(fd221)*], WY1381 [*nekl-2(fd91); fdEx278 (pDF166 [nekl-2(+)]; pTG96); sajIs31 (lin-26p*::*wsp-1[crib]*::*mCherry; unc-119R)*], WY1382 [*nekl-3(sv3) X; mnEx174 (F19H6; pTG96); sajIs31 (lin-26p*::*wsp-1[crib]*::*mCherry; unc-119R)*], WY1385 [*fbn-1(tm290); fdEx250(pMH281*, *sur-5*::*rfp); sajIs31 (lin-26p*::*wsp-1[crib]*::*mCherry; unc-119R)*], WY1422 [*nekl-2(fd91); unc-119(ed3); fdEx278 (pDF166 [nekl-2(+)]; pTG96); xnIs506(cdc42p*::*gst*::*gfp*::*wsp-1[gbd]; unc-119[+])*], and WY1424 [*nekl-3(sv3)X; unc-119(ed3); mnEx174(F19H6; pTG96); xnIs506(cdc42p*::*gst*::*gfp*::*wsp-1[gbd]; unc-119[+])*].

### RNA interference

RNAi was performed using bacterial strains from Geneservice using standard protocols [95]. In the study that identified *cdc-42*, we screened all available clones on LGII (~3000 clones) for clones that allowed *nekl-3(sv3)* mutants to bypass L2/L3 arrest. Control RNAi experiments were carried out using the bacterial strain HT115 carrying either a vector plasmid (pPD129.36) or *gfp(RNAi)*. Marker localization studies involving *mlt-3(RNAi)* were carried out using strains that were previously grown for several generations on *lin-35(RNAi)* plates, which increases RNAi sensitivity [96]. Combined RNAi treatments in S2 Fig were performed using RNAi-hypersensitive *rrf-3(pk1426)* mutants. Bacteria expressing *mlt-2*, *mlt-3*, *cdc-42*, and *gfp(RNAi)* constructs were grown to the same density and combined at specific ratios. A bacterial culture expressing *mlt-2(RNAi)* was combined at a 2:1 ratio with bacteria expressing *cdc-42(RNAi)* or *gfp(RNAi)*, whereas the *mlt-3(RNAi)* bacterial strain was mixed with the above RNAi clones at a 1:2 ratio.

### CDC-42 gain-of-function mutants

Plasmids were generated by amplifying the *dpy-7* promoter region from plasmid pCFJ1662 (gift of Barth Grant) using the primers 5’- tcgacaaagctttctcattccacgatttctc-3’ and 5’- tcgacactgcagttatctggaacaaaatgtaagaa-3’. Following digestion with HindIII and PstI, PCR product was inserted into pD95.75 to create plasmids pDF400. Genomic *cdc-42* coding sequences was amplified from fosmids *WRM069cE02* and *WRM0610aC01* using primers 5’-tgattcggatccatgcagacgatcaagtgcgtc-3’ and 5’-tgattcggtacctcgttccaattcacccactca-3’, digested with BamHI and KpnI, and ligated into PDF400 to produce pDF406. G12V and Q61L *cdc-42* mutants were generated using the Q-5 Site-Directed Mutagenesis Kit (New England Biolabs) and the following primers: For G12V primers 5’-gttggagatgtagctgtcggtaaaac-3’ and 5’-gacgacgcacttgatcgt-3’; for Q61L primers 5’-actgctggactggaagattac-3’ and 5’- atcaaacaatcctaatgtgtatg-3’. G12V (pDF409) and Q61L (pDF412) Plasmids were sequence confirmed and injected with *sur-5*::GFP (pTG96) into 20–25 N2 worms at the following concentrations: 50 ng/μl *sur-5*::*GFP* + 100 ng/μl *cdc-42*, 100 ng/μl *sur-5*::*GFP* + 50 ng/μl *cdc-42*, 100 ng/μl *sur-5*::*GFP* + 20 ng/μl *cdc-42*, 100 ng/μl *sur-5*::*GFP* + 15 ng/μl *cdc-42*. Each injected worm was transferred to a new plate every 24 hours and its progeny were scored and imaged.

### Genetic identification and analysis of *sid-3*

*sid-3(fd139)* was identified as described in Joseph et al. [63]. CRISPR/Cas9 technology was used to generate additional loss-of-function alleles of *sid-3* in the *nekl-2 (fd81); nekl-3(gk894345)* background. Specifically, genome engineering was performed using CRISPR/Cas9 ribonucleoproteins in combination with the *dpy-10* co-CRISPR method [97–99]. crRNAs were designed to target sequences in the N terminus (5’-AGTGCTCCGCAAAGCACAGT-3’) or C terminus (5’-TGAGCCGATTCTCTCGTCTG-3’) of *sid-*3. Absence of repair templates allowed for non-homologous repair mechanisms to generate random frameshifts and premature stop codons in the desired regions. Six loss-of-function alleles were isolated, including *fd213*, a 16-bp insertion and one point mutation in exon 13, causing a frameshift after L987 and premature stop at amino acid position 1034; *fd214*, a 7-bp deletion in exon 13, causing a frameshift after L987 and premature stop at amino acid position 1006; *fd218*, a 5-bp deletion in exon 1, causing a frameshift after K19 and premature stop at amino acid position 25; *fd219*, a 1-bp deletion in exon 1, causing a frameshift after A20 and premature stop at amino acid position 101; and *fd221*, a 31-bp insertion and 1-bp deletion in exon 1, causing a frameshift after A20 and premature stop at amino acid position 24 (also see S2 Table). Suppressed *nekl* mutants were analyzed at the last molt (Fig 6) or as L4 or adult worms (Figs 5 and 8).

### Actin staining

Actin staining was performed following a modified procedure from Costa et al. [64]. Animals were washed in M9 solution, and fixation was carried out by incubating animals in fixative (2% paraformaldehyde in 0.05 M Na_3_PO_4,_ pH 7.4) for 2 h at room temperature. Worms were washed three times in PBS and stored at 4°C. Phalloidin-TRITC (0.1 mg/ml, dissolved in methanol) was diluted in permeabilization solution (0.05% Triton X-100, 0.1 M NaCl, 3.7% sucrose) at a ratio of 1:500. Equal amounts of liquid containing fixed animals and the phalloidin-containing solution were mixed and incubated in a dark chamber for at least 1 h. To avoid possible post mortem artifacts in our analyses, we scored only the animals that did not show any alterations in the morphology of their internal organs.

### Microscopy

Fluorescent images were acquired using an Olympus IX81 inverted microscope with a spinning-disc confocal head (CSUX1 Yokogawa). Confocal illumination was provided by an ILE-4 laser launch (SpectralAppliedResearch). MetaMorph7.7 software was used for image acquisition. DIC images were acquired using a Nikon Eclipse epifluorescence microscope and Open Lab software. This setup was also used for measuring body length and width. Body width was measured in the central region of animals; 50 animals were scored for each category. Animals were immobilized using a 0.1 M solution of levamisole. Distinguishing molting larvae from intermolt larvae was achieved using 0.5-μm red beads mixed with food (Sigma L3280), following the protocol of Nika et al. [100]. For Fig 6, wild-type molting and intermolt animals were categorized using a *mlt-10p*::*gfp-pest* reporter [101, 102].

Fluorescence intensity of GFP::CDC-42 in Fig 3 was measured using the FIJI program. For each animal, average fluorescence intensity was measured in 10 equal, randomly selected regions of hyp7 and average values were determined. GFP::CDC-42 was analyzed in 27 molting and 30 intermolt wild-type animals. Fluorescence intensity of GST-GFP::WSP-1(GBD) in Fig 4 was measured using ImageJ program. Mean intensities in selected areas were calculated for 11 animals or more and averaged. Background mean intensities were subtracted. Average area of puncta in S5 Fig and number of puncta in S10 Fig were calculated using FIJI program, using ten animals for each genetic background. Colocalization analyses were performed on L4-stage larvae to allow for better visualization of fluorescent structures. Line scanes in S3 and S9 Figs were made using Metamorph. Colocalization with VAB-10(ABD)::mCherry marker was performed in apical regions of epidermis that are slightly more medial than the plane containing actin bundles, as this region showed the greatest extent of colocalization. Transverse plane reconstructions of GFP::CHC-1 morphology were created using the FIJI program 3D projection function in selected 1000-pixel × 7-pixel regions of 24-frame *z*-stacks. Distances between neighboring planes in each *z*-stack were 0.2 μm. Composite sagittal plane projections were created from the same *z*-stacks in FIJI, using the Temporal-Color Code function.

## Supporting information

S1 Fig *cdc-42(RNAi)* increases the width of *nekl* null mutants. (A,B) Graphic representation of body measurements of *nekl-3(gk506)* (A) and *nekl-2(gk839)* (B) animals grown on control RNAi (blue dots) and *cdc-42(RNAi)* (orange dots). Body width and body length are indicated in micrometers on the *x* and *y* axis, respectively. Each dot represents one animal (n = 50 for each genotype and RNAi treatment). A Student’s t-test was used to analyze differences in width.
(TIF)

S2 Fig *mlt-2(RNAi)* and *mlt-3(RNAi)* molting defects are partially suppressed by *cdc-42(RNAi)*. (A,B) Graphic representations of body measurements of *rrf-3(pk1426)* RNAi-hypersensitive animals treated with *mlt-2(RNAi)* (A) or *mlt-3(RNAi)* (B) combined with either control RNAi (blue dots) or *cdc-42(RNAi)* (orange dots). Each dot in graphs A and B represents one animal (n = 50 for each RNAi treatment). In both graphs, orange dots are typically shifted to the right side, indicating that *cdc-42(RNAi)* results in a body width increase. A Student’s t-test was used to analyze differences in width.
(TIF)

S3 Fig Analysis of GFP::CDC-42 colocalization with NEKL–MLT components. (A-C) Line scans of selected green (NeonGreen) and red (mKate2) puncta in representative images from Fig 3. Fluorescence intensity peaks indicate high colocalization between GFP::CDC-42 and MLT-2::mKate2 at the seam cell boundary in apical planes (A) and throughout the epidermis in subapical planes (B). (C) NEKL-3::mKate2 shows some colocalization with GFP::CDC-42 in the apical plane. The *x* axis represents fluorescence intensity (gray level) in arbitrary units; *y* axis represents distance from the starting point of line scan in micrometers. Each line scan starts at the upper left corner; direction of each line scan is indicated by the arrowhead. Bar size in C = 5 μm in A–C.
(TIF)

S4 Fig Inhibition of *mlt-3* deregulates GFP::CDC-42 localization in the epidermis. (A) GFP::CDC-42 is expressed in dispersed puncta throughout the epidermis in animals treated with a control RNAi. (B) GFP::CDC-42 is mislocalized in *mlt-3(RNAi)* animals, resulting in the formation of large aggregates (white arrows). (C,D) Molting-defective *mlt-11(RNAi)* (C) and *qua-1(RNAi)* (D) animals display a relatively normal pattern of GFP::CDC-42 localization. Bar size in D = 10 μm in A–D.
(TIF)

S5 Fig NEKL-2 and NEKL-3 control CDC-42 localization. (A–H) Wild-type expression of a reporter for active CDC-42 (WSP-1(CRIB)::mCherry) (A) is changed in *nekl-2(fd91)* (B) and *nekl-3(sv3)* (C) mutants, unlike in *fbn-1(tm290)* molting-defective controls (E). WSP-1(CRIB)::mCherry puncta are more numerous in *nekl* mutants (lower triangles in B and C) and sometimes form large aggregates (upper triangles in B and C). Bar size in D = 10 μm in A–D. (E) Quantitative comparison of average area of WSP-1(CRIB)::mCherry puncta measured in ten randomly selected animals for each indicated stage and genetic background. Error bars represent standard deviations. *p* values were derived using a Student's t-test.
(TIF)

S6 Fig Molting defects induced by the expression of constitutively activated CDC-42 variants. (A–K) Examples of molting defects in larvae expressing either the G12V or Q61L hyperactive variants of CDC-42 in the epidermis. DIC overlay (A, C, E, G, I, K) and accompanying GFP (B, D, F, H, J) images (panel K does not have an accompanying GFP image). DIC images are overlaid with GFP (colored magenta). Inset panels are indicated by colored boxes. Panels A–D were taken from the same animal. Abnormal cuticle is indicated by arrowheads. Bar sizes = 20 μm.
(TIF)

S7 Fig Examples of phalloidin staining of *nekl* mutants. (A-D) Images of phalloidin staining in wild type (A), *nekl-2(fd91)* (B), *nekl-3(sv3)* (C) and *nekl-2(fd81); nekl-3(gk894345)* (D) animals. Wild type animals shown in A are the same animals from the main figure Fig 6. Orange arrows indicate areas where formation of actin parallel rows is initiated in the epidermis. Red asterisks indicate areas of high phalloidin fluorescence from underlying body wall muscles. White arrows in D indicate region of body constriction by old cuticle. Bar size in D = 10 μm in A–D.
(TIF)

S8 Fig Actin morphology in the epidermis of *cdc-42(gk388)* molting-defective animals. (A-F) Phalloidin staining of *cdc-42(gk388)* larvae with molting defects. Wild type animals shown in A and B are the same animals from the main figure Fig 6. Some animals form parallel rows of actin puncta in portions of the epidermis (C and D), which are somewhat similar to actin bundles in wild type molting animals (B). Other animals show apical actin phenotypes atypical for molting animals (E and F), which are more similar to intermolt patterns in wild type (A). Bar size in F = 10 μm in A–F.
(TIF)

S9 Fig Analysis of VAB-10(ABD)::mCherry colocalization with NEKL–MLT components. (A-C) Line scans of selected green (NeonGreen and GFP) and red (mCherry) puncta in representative images from Fig 7. Subapical region of epidermis was analyzed. Fluorescence intensity peaks indicate colocalization of VAB-10(ABD)::mCherry with NEKL-2::NeonGreen (A), MLT-4::GFP (B), and NEKL-3::NeonGreen (C). *x* axis represents fluorescence intensity (gray level) in arbitrary units; *y* axis represents distance from the starting point of line scan in micrometers. Each line scan starts at the upper left corner; direction of each line scan is indicated by the arrowhead. Bar size in C = 10 μm in A–C.
(TIF)

S10 Fig Quantitative analysis of GFP::CHC-1 puncta abundance. Graphic representation of average number of GFP::CHC-1 puncta per 100 μm^2^ at the apical surface of hyp7 measured in ten randomly selected animals for each indicated genomic background and feeding condition. Error bars represent standard deviations. *p* values were derived using a student’s t-test.
(TIF)

S11 Fig Examples of endocytosis defects in *nekl-2(fd81); nekl-3(gk894345)* animals following CDC-42 and SID-3 depletion. Wild type animal shown in A is the same as in the Fig 6B. (B) Example of a *nekl-2(fd81); nekl-3(gk894345); cdc-42(RNAi)* animal that was superficially suppressed for molting defects but showed gross mislocalization of GFP::CHC-1. (C) Partial mislocalization of GFP::CHC-1 in a suppressed *nekl-2(fd81); nekl-3(gk894345); sid-3(fd139)* triple mutant. Color scale is shown in Fig 8A. Bar sizes in C = 10 μm (horizontal) and 2 μm (vertical) in A–C.
(TIF)

S1 Table List of fluorescently tagged genes used in this study. (DOCX)

S2 Table Mutated regions of CRISPR/Cas9-generated *sid-3* lines. Alignments of wild-type sequence and corresponding mutated regions in *sid-3(fd213)*, *sid-3(fd214)*, *sid-3(fd218)*, *sid-3(fd219)*, and *sid-3(fd221)* mutants. Mutated regions are flanked upstream and downstream with ten wild-type nucleotides. Deleted regions are marked in red; insertions are indicated in blue. The number of the first nucleotide in each sequence, based on *sid-3* isoform a, is shown in parentheses.
(DOCX)

S1 File Contains raw data for Fig 1, Fig 3, Fig 4, Fig 5, S1 Fig, S2 Fig, S5 Fig and S10 Fig. (XLSX)
