# Supplementary figures and images for: Actin organization and endocytic trafficking are controlled by a network linking NIMA-related kinases to the CDC-42-SID-3/ACK1 pathway
Source: PLoS Genet. 2018 Apr 2;14(4):e1007313. doi: 10.1371/journal.pgen.1007313 (PMC5897031; doi:10.1371/journal.pgen.1007313)

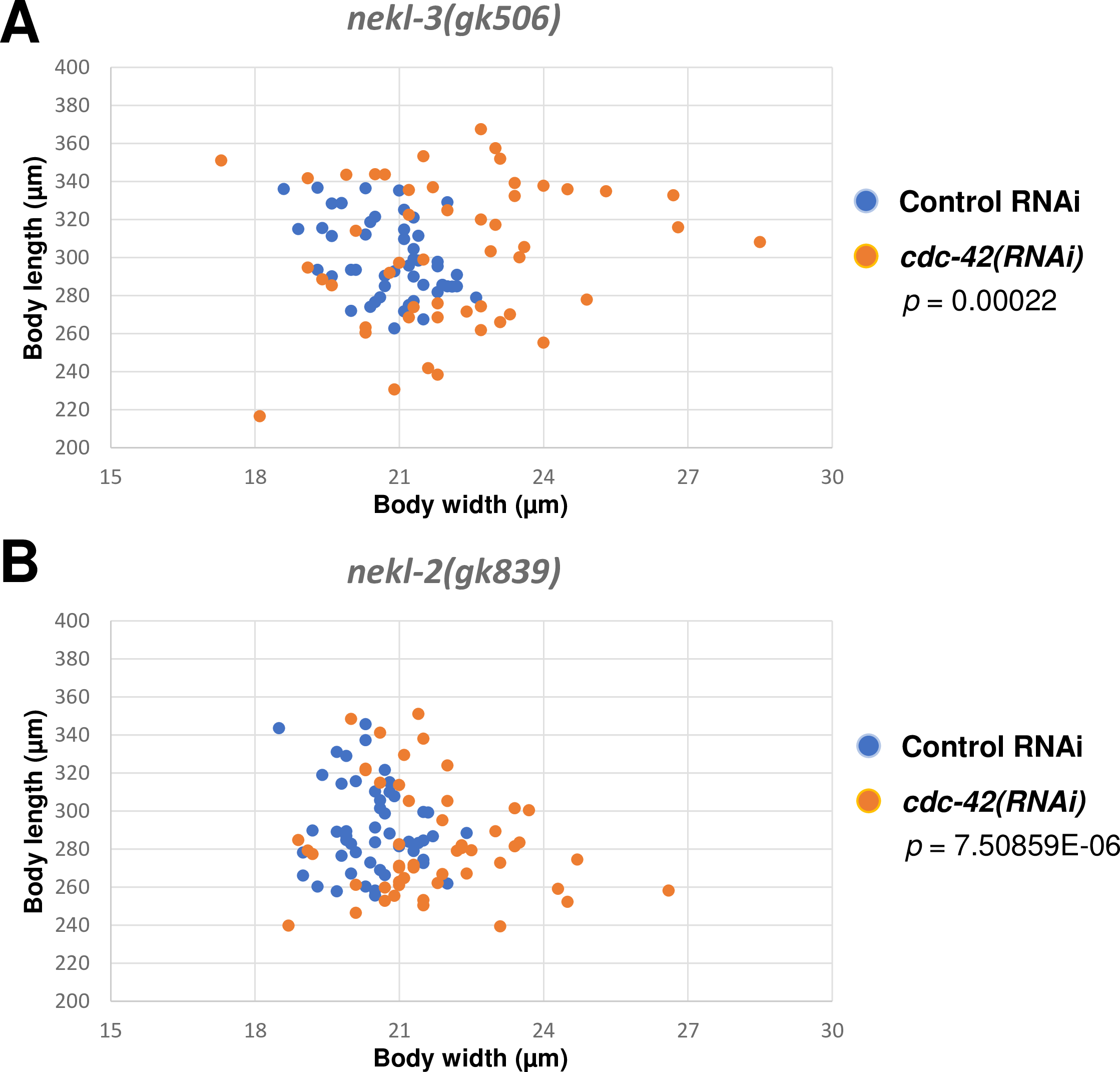

Supplement: S1 Fig — (A,B) Graphic representation of body measurements of nekl-3(gk506) (A) and nekl-2(gk839) (B) animals grown on control RNAi (blue dots) and cdc-42(RNAi) (orange dots). Body width and body length are indicated in micrometers on the x and y axis, respectively. Each dot represents one animal (n = 50 for each genotype and RNAi treatment). A Student’s t-test was used to analyze differences in width. (TIF) [file pgen.1007313.s001.tif]

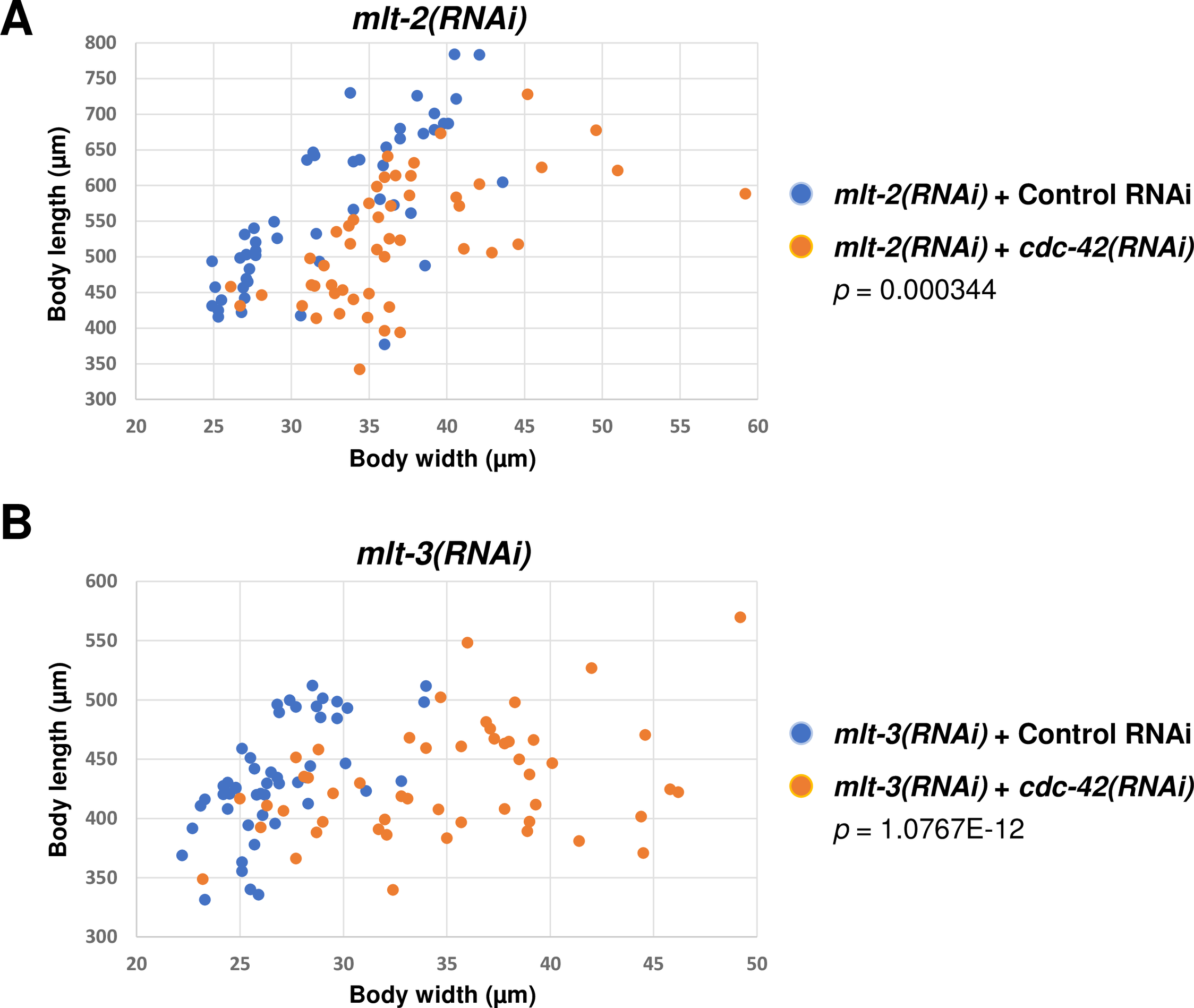

Supplement: S2 Fig — (A,B) Graphic representations of body measurements of rrf-3(pk1426) RNAi-hypersensitive animals treated with mlt-2(RNAi) (A) or mlt-3(RNAi) (B) combined with either control RNAi (blue dots) or cdc-42(RNAi) (orange dots). Each dot in graphs A and B represents one animal (n = 50 for each RNAi treatment). In both graphs, orange dots are typically shifted to the right side, indicating that cdc-42(RNAi) results in a body width increase. A Student’s t-test was used to analyze differences in width. (TIF) [file pgen.1007313.s002.tif]

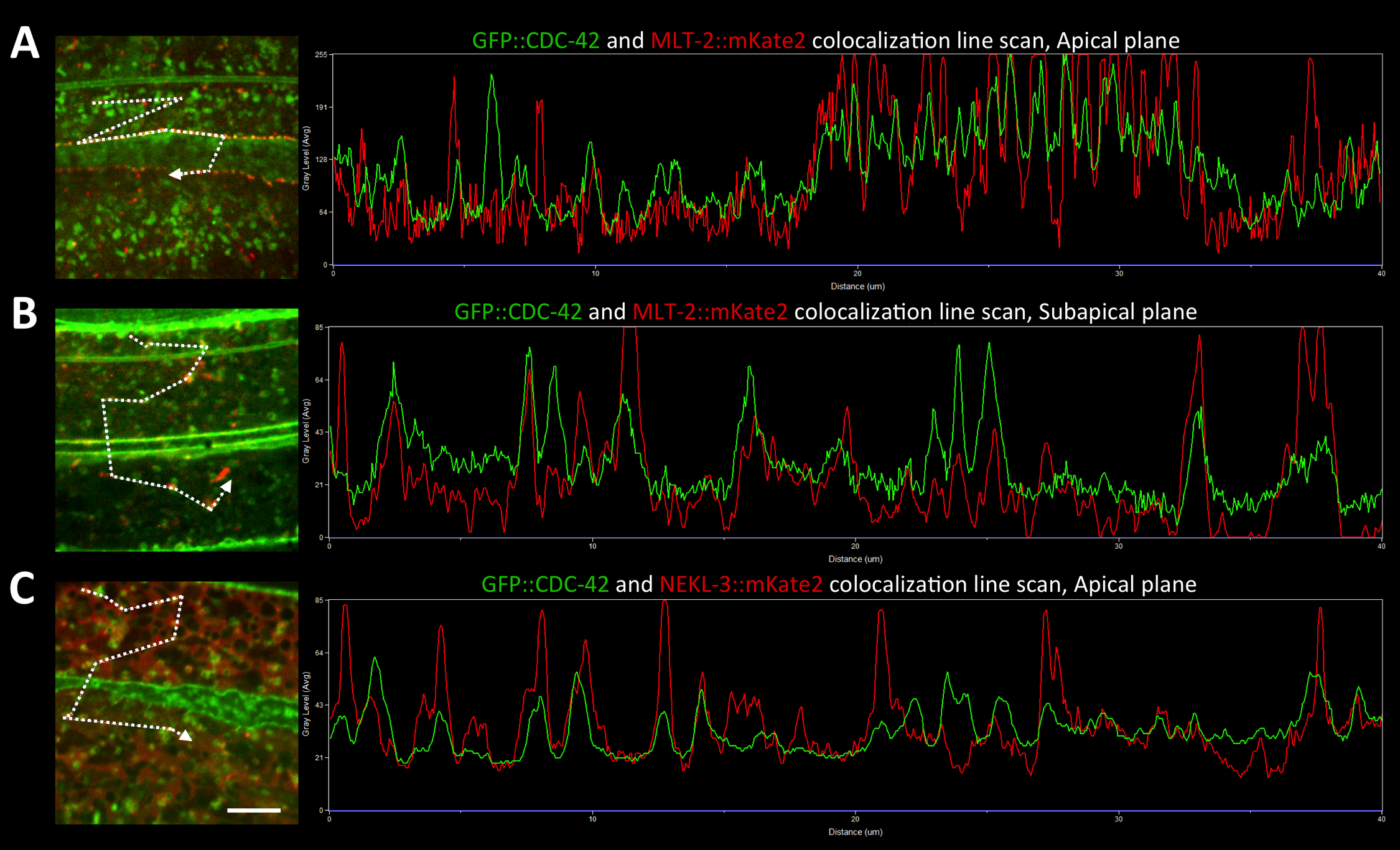

Supplement: S3 Fig — (A-C) Line scans of selected green (NeonGreen) and red (mKate2) puncta in representative images from Fig 3. Fluorescence intensity peaks indicate high colocalization between GFP::CDC-42 and MLT-2::mKate2 at the seam cell boundary in apical planes (A) and throughout the epidermis in subapical planes (B). (C) NEKL-3::mKate2 shows some colocalization with GFP::CDC-42 in the apical plane. The x axis represents fluorescence intensity (gray level) in arbitrary units; y axis represents distance from the starting point of line scan in micrometers. Each line scan starts at the upper left corner; direction of each line scan is indicated by the arrowhead. Bar size in C = 5 μm in A–C. (TIF) [file pgen.1007313.s003.tif]

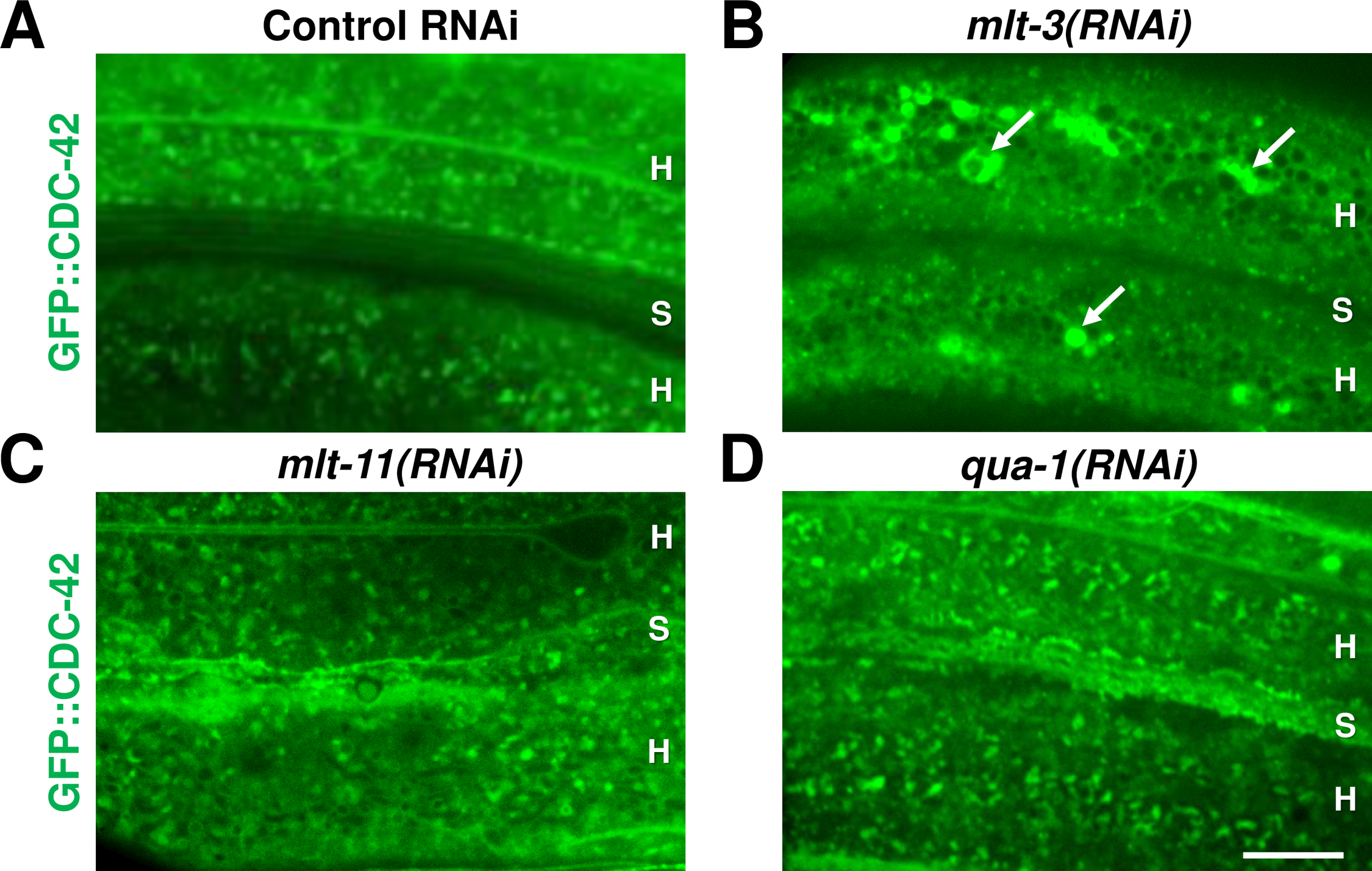

Supplement: S4 Fig — (A) GFP::CDC-42 is expressed in dispersed puncta throughout the epidermis in animals treated with a control RNAi. (B) GFP::CDC-42 is mislocalized in mlt-3(RNAi) animals, resulting in the formation of large aggregates (white arrows). (C,D) Molting-defective mlt-11(RNAi) (C) and qua-1(RNAi) (D) animals display a relatively normal pattern of GFP::CDC-42 localization. Bar size in D = 10 μm in A–D. (TIF) [file pgen.1007313.s004.tif]

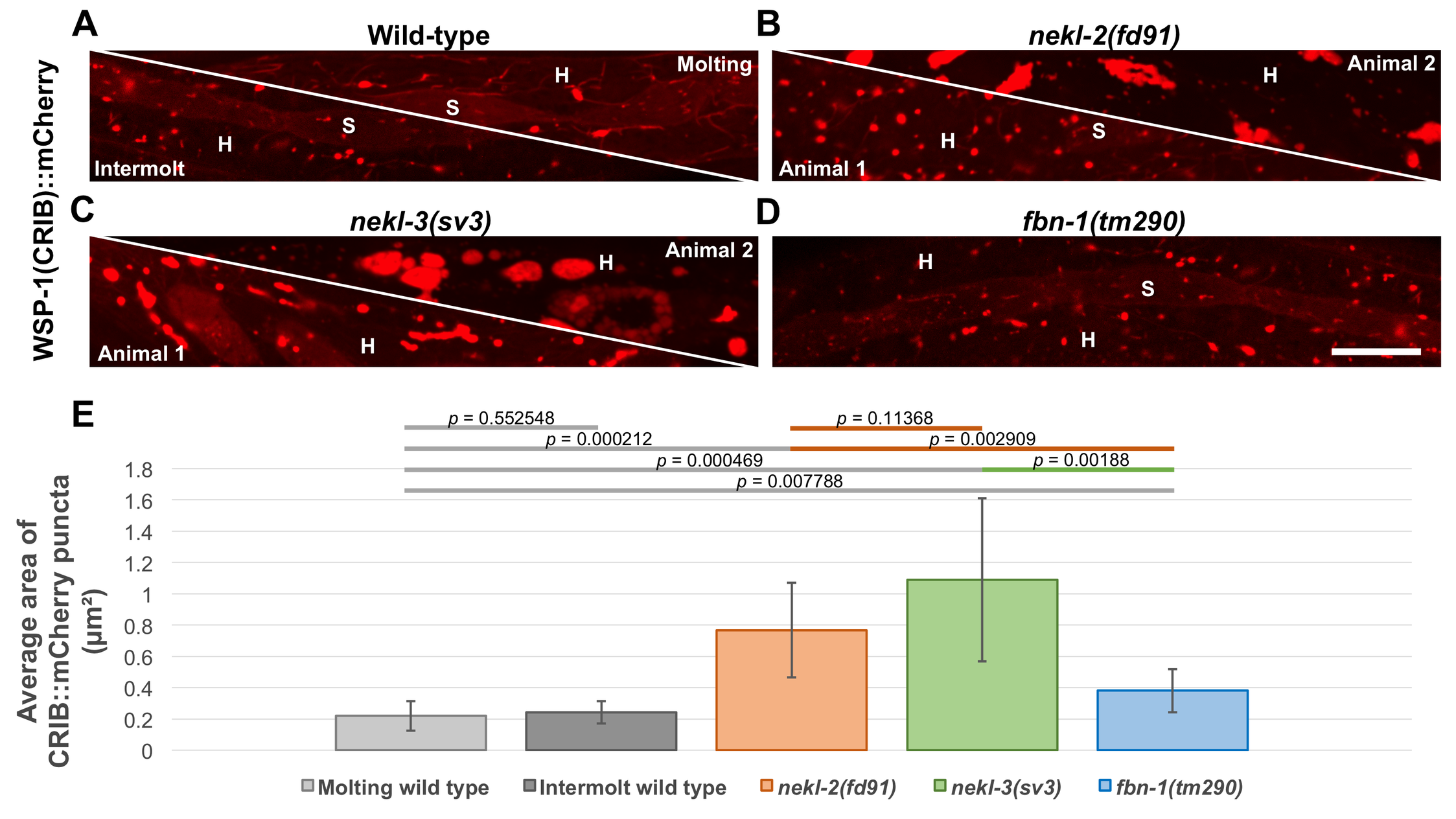

Supplement: S5 Fig — (A–H) Wild-type expression of a reporter for active CDC-42 (WSP-1(CRIB)::mCherry) (A) is changed in nekl-2(fd91) (B) and nekl-3(sv3) (C) mutants, unlike in fbn-1(tm290) molting-defective controls (E). WSP-1(CRIB)::mCherry puncta are more numerous in nekl mutants (lower triangles in B and C) and sometimes form large aggregates (upper triangles in B and C). Bar size in D = 10 μm in A–D. (E) Quantitative comparison of average area of WSP-1(CRIB)::mCherry puncta measured in ten randomly selected animals for each indicated stage and genetic background. Error bars represent standard deviations. p values were derived using a Student's t-test. (TIF) [file pgen.1007313.s005.tif]

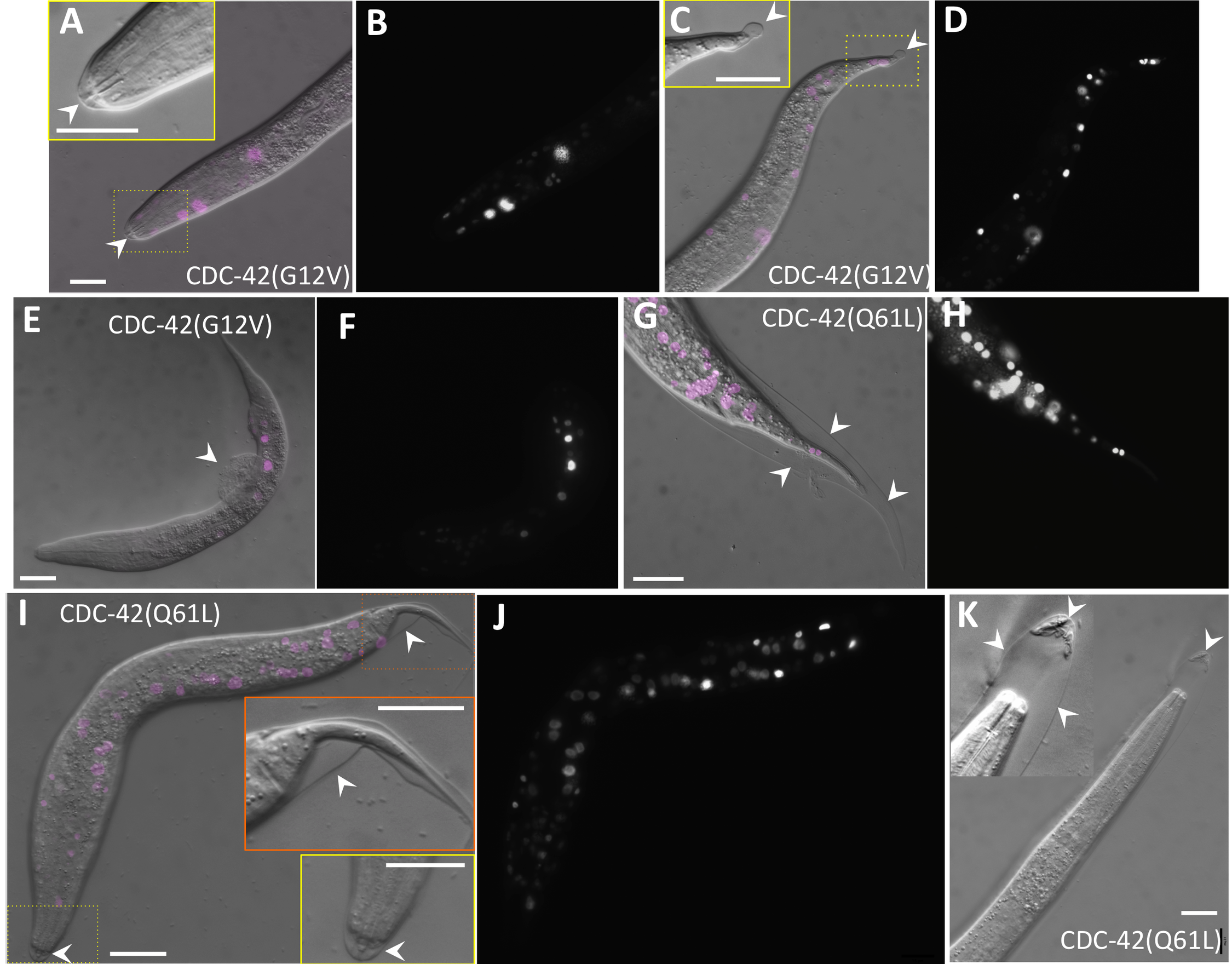

Supplement: S6 Fig — (A–K) Examples of molting defects in larvae expressing either the G12V or Q61L hyperactive variants of CDC-42 in the epidermis. DIC overlay (A, C, E, G, I, K) and accompanying GFP (B, D, F, H, J) images (panel K does not have an accompanying GFP image). DIC images are overlaid with GFP (colored magenta). Inset panels are indicated by colored boxes. Panels A–D were taken from the same animal. Abnormal cuticle is indicated by arrowheads. Bar sizes = 20 μm. (TIF) [file pgen.1007313.s006.tif]

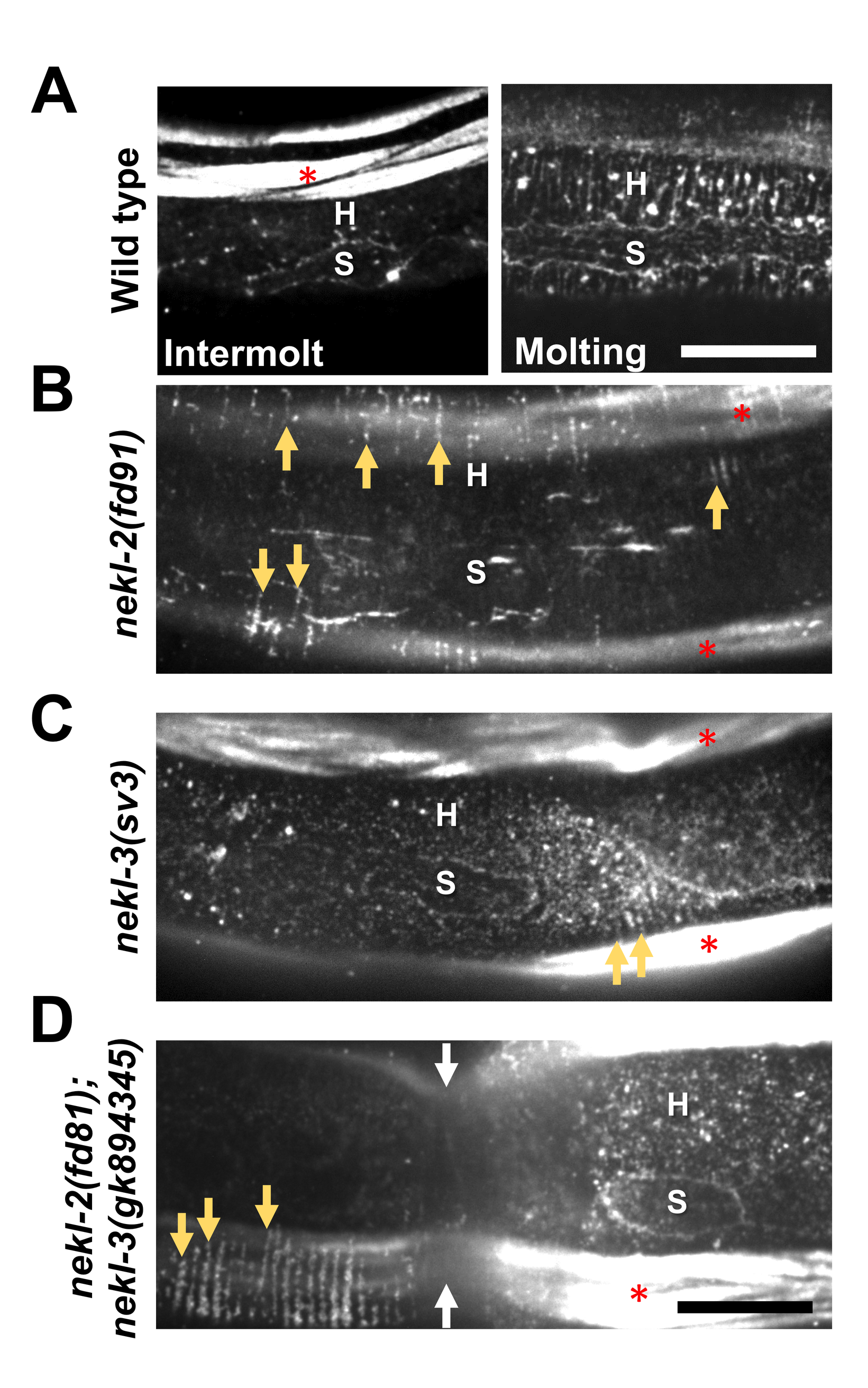

Supplement: S7 Fig — (A-D) Images of phalloidin staining in wild type (A), nekl-2(fd91) (B), nekl-3(sv3) (C) and nekl-2(fd81); nekl-3(gk894345) (D) animals. Wild type animals shown in A are the same animals from the main figure Fig 6. Orange arrows indicate areas where formation of actin parallel rows is initiated in the epidermis. Red asterisks indicate areas of high phalloidin fluorescence from underlying body wall muscles. White arrows in D indicate region of body constriction by old cuticle. Bar size in D = 10 μm in A–D. (TIF) [file pgen.1007313.s007.tif]

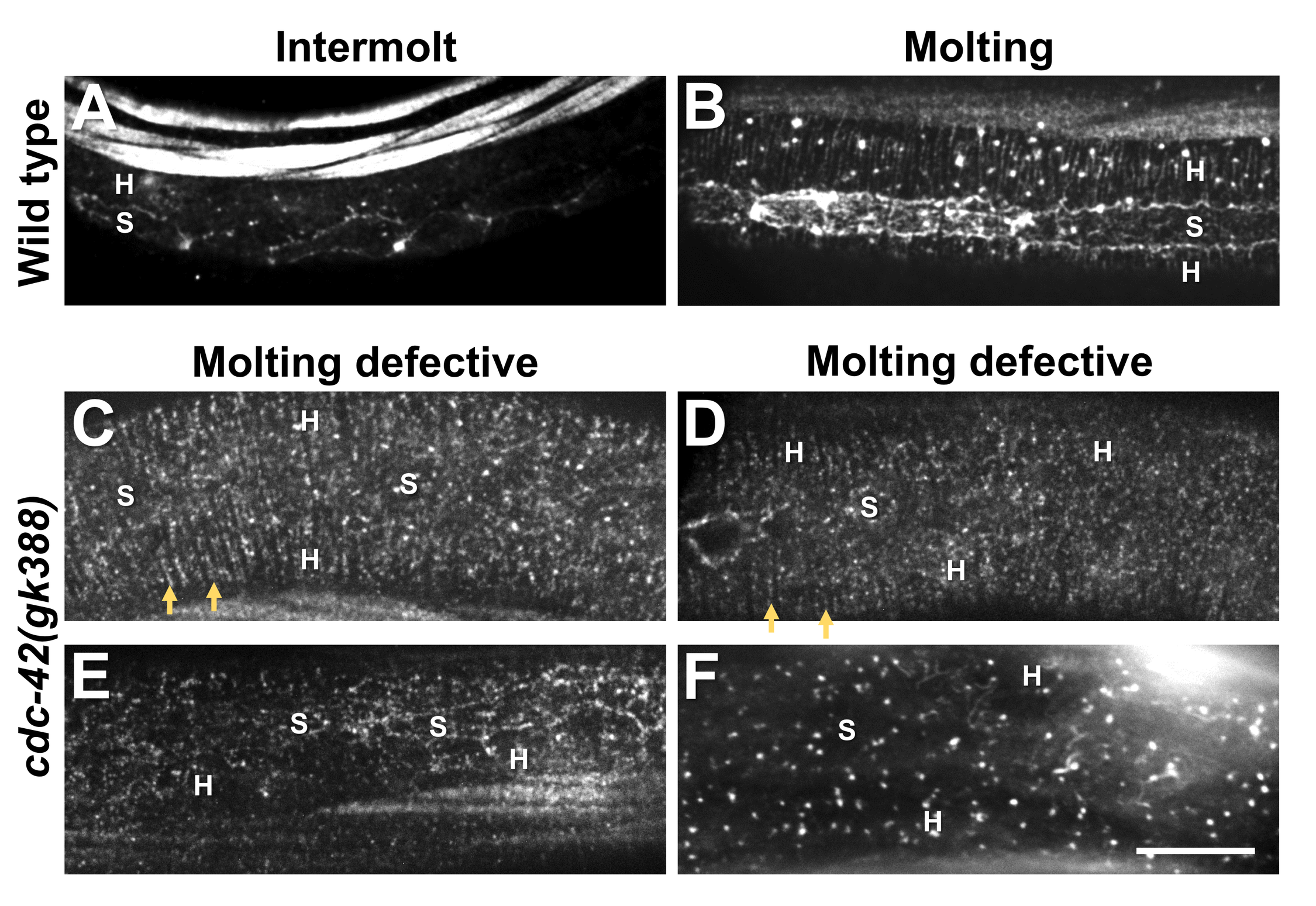

Supplement: S8 Fig — (A-F) Phalloidin staining of cdc-42(gk388) larvae with molting defects. Wild type animals shown in A and B are the same animals from the main figure Fig 6. Some animals form parallel rows of actin puncta in portions of the epidermis (C and D), which are somewhat similar to actin bundles in wild type molting animals (B). Other animals show apical actin phenotypes atypical for molting animals (E and F), which are more similar to intermolt patterns in wild type (A). Bar size in F = 10 μm in A–F. (TIF) [file pgen.1007313.s008.tif]

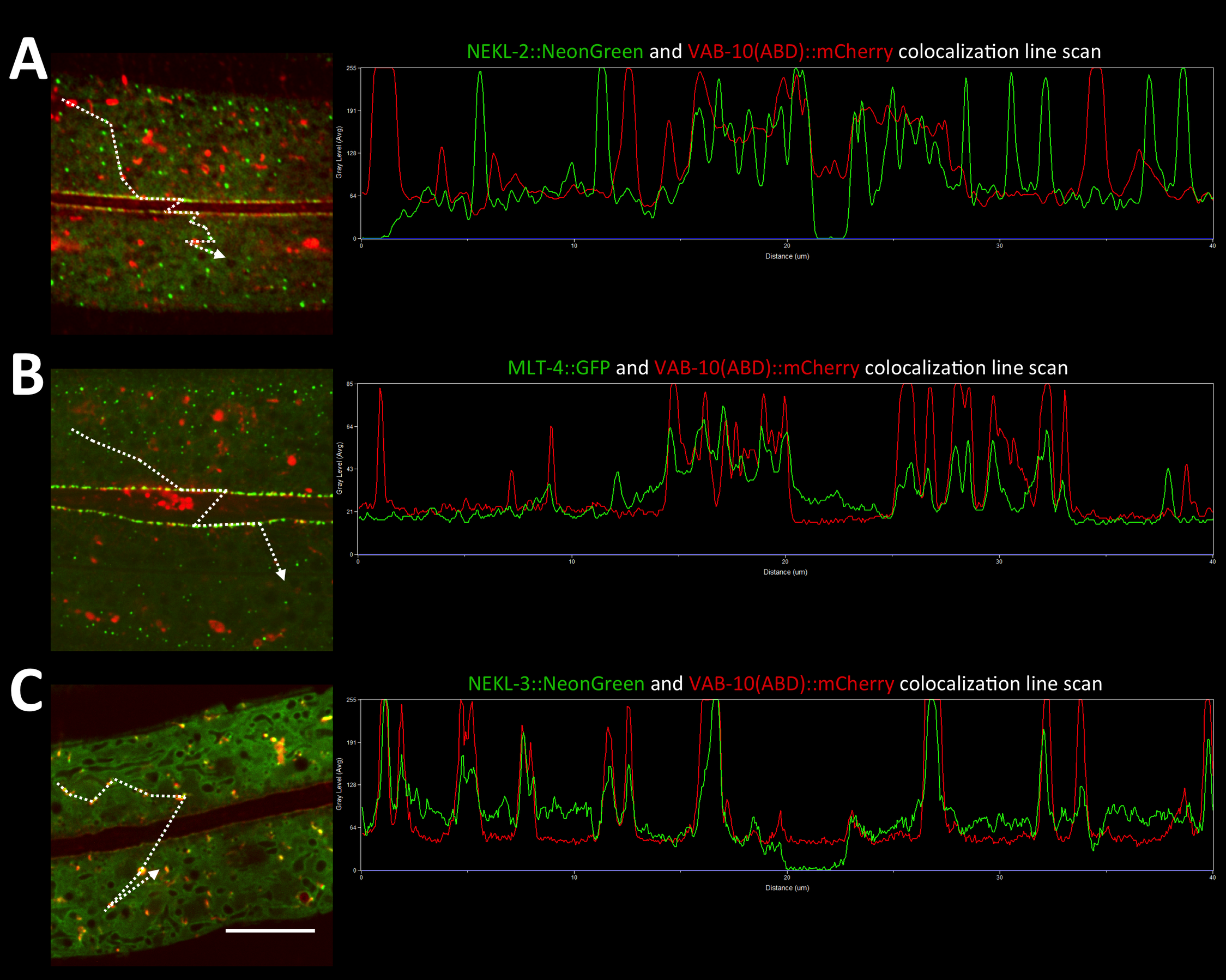

Supplement: S9 Fig — (A-C) Line scans of selected green (NeonGreen and GFP) and red (mCherry) puncta in representative images from Fig 7. Subapical region of epidermis was analyzed. Fluorescence intensity peaks indicate colocalization of VAB-10(ABD)::mCherry with NEKL-2::NeonGreen (A), MLT-4::GFP (B), and NEKL-3::NeonGreen (C). x axis represents fluorescence intensity (gray level) in arbitrary units; y axis represents distance from the starting point of line scan in micrometers. Each line scan starts at the upper left corner; direction of each line scan is indicated by the arrowhead. Bar size in C = 10 μm in A–C. (TIF) [file pgen.1007313.s009.tif]

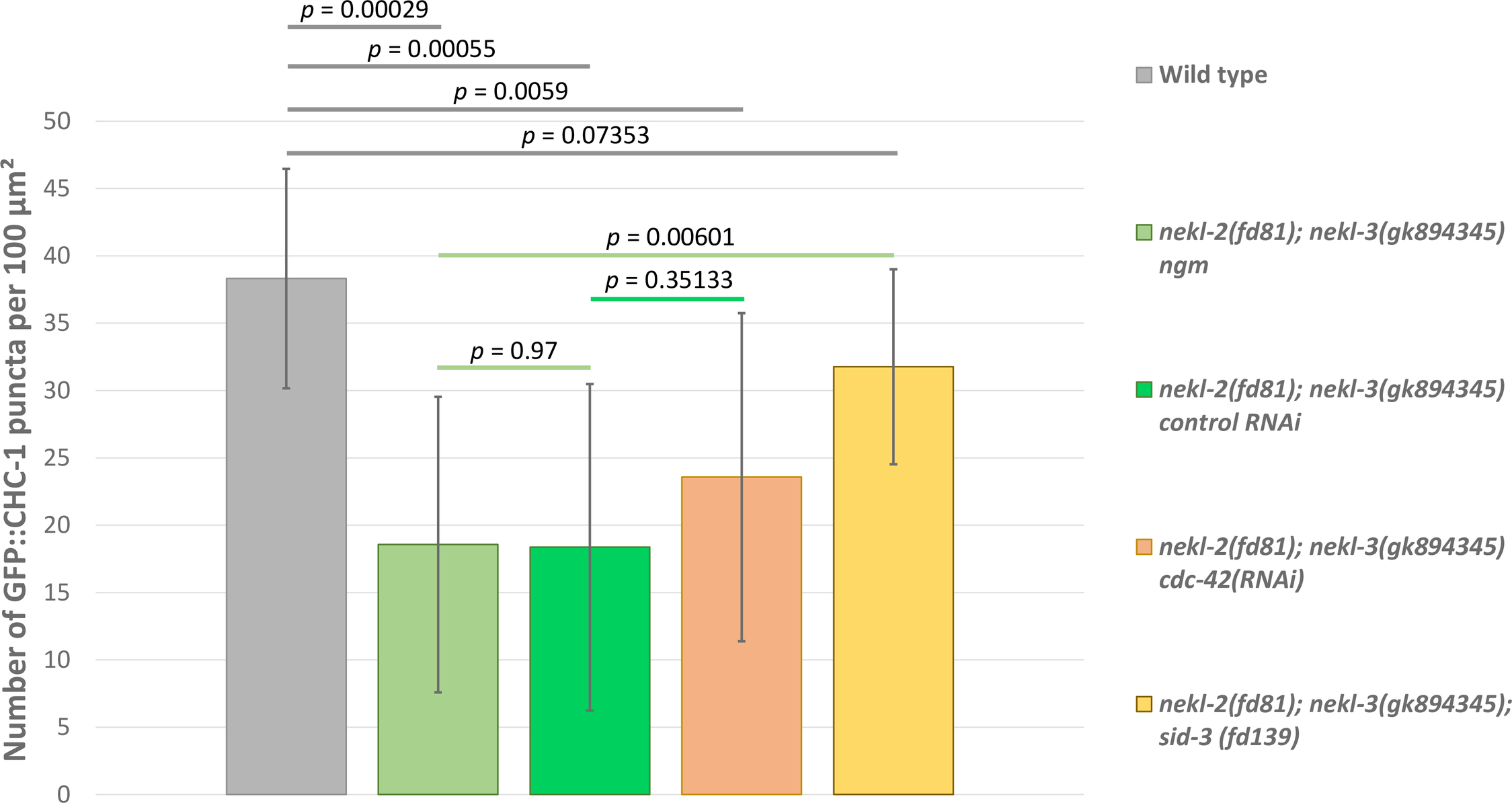

Supplement: S10 Fig — Graphic representation of average number of GFP::CHC-1 puncta per 100 μm2 at the apical surface of hyp7 measured in ten randomly selected animals for each indicated genomic background and feeding condition. Error bars represent standard deviations. p values were derived using a student’s t-test. (TIF) [file pgen.1007313.s010.tif]

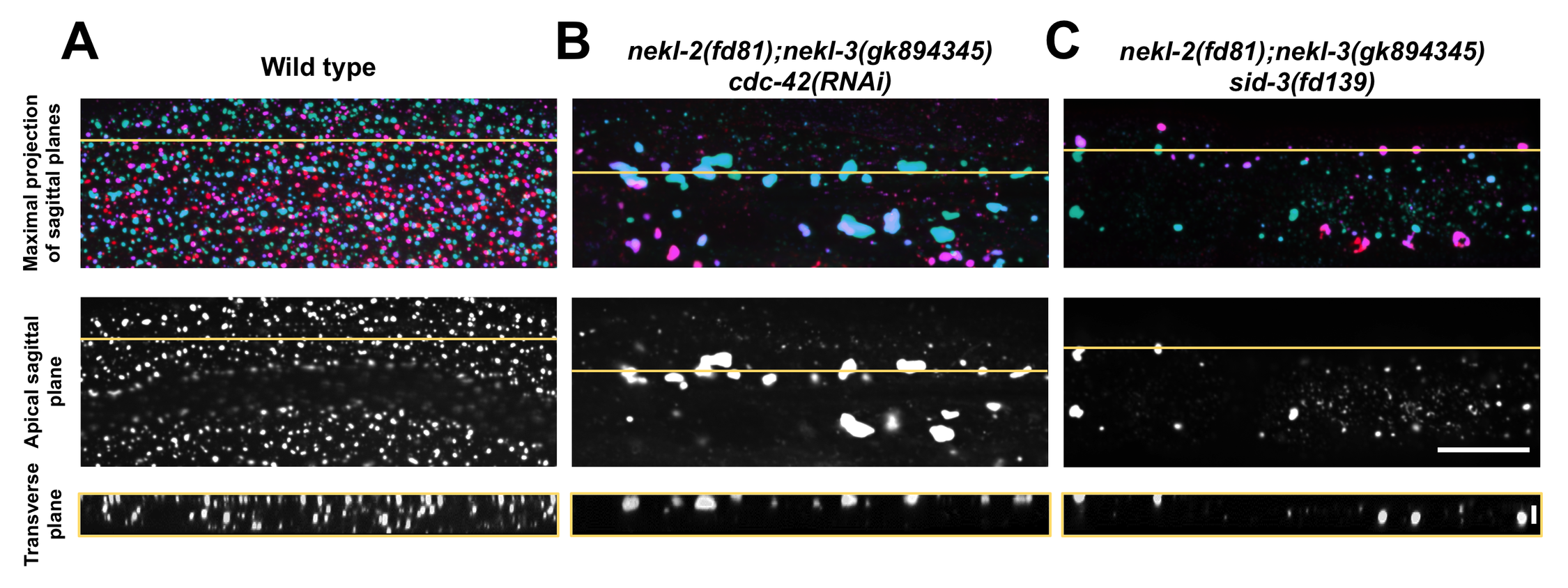

Supplement: S11 Fig — Wild type animal shown in A is the same as in the Fig 6B. (B) Example of a nekl-2(fd81); nekl-3(gk894345); cdc-42(RNAi) animal that was superficially suppressed for molting defects but showed gross mislocalization of GFP::CHC-1. (C) Partial mislocalization of GFP::CHC-1 in a suppressed nekl-2(fd81); nekl-3(gk894345); sid-3(fd139) triple mutant. Color scale is shown in Fig 8A. Bar sizes in C = 10 μm (horizontal) and 2 μm (vertical) in A–C. (TIF) [file pgen.1007313.s011.tif]
