# Supplementary material for: Actin organization and endocytic trafficking are controlled by a network linking NIMA-related kinases to the CDC-42-SID-3/ACK1 pathway
Source: PLoS Genet. 2018 Apr 2;14(4):e1007313. doi: 10.1371/journal.pgen.1007313 (PMC5897031; doi:10.1371/journal.pgen.1007313)
Supplement: S1 Table — (DOCX) [file pgen.1007313.s012.docx]

|  | **Tagged gene/domain** | **Allele name** | **Fluorescent tag** | **Integration** | **Functionality, copy number** | **Expressing strains** | **References** |
| --- | --- | --- | --- | --- | --- | --- | --- |
| 1 | *cdc-42* | *opIs295* | *gfp* | Integrated, multicopy | Functional | WS5018, WY1337, WY1338, WY1360, WY1361, WY1376 | (1, 2) |
| 2 | *chc-1* | *pwIs528* | *gfp* | Integrated, multicopy | Not tested | RT1378, WY1242, WY1345 | (3, 4) |
| 3 | *mlt-2* | *fd95* | *mKate2* | Integrated, endogenous locus | Functional | WY1337 | (3) |
| 4 | *mlt-4* | *fd114* | *gfp* | Integrated, endogenous locus | Functional | WY1333 | (3) |
| 5 | *nekl-2* | *fd100* | *NeonGreen* | Integrated, endogenous locus | Functional | WY1331 | (3) |
| 6 | *nekl-3* | *fd106* | *mKate2* | Integrated, endogenous locus | Functional | WY1338 | (3) |
|  |  | *fd118* | *NeonGreen* |  | Functional | WY1332 | (3) |
| 7 | *vab-10(abd)* | *mcIs40* | *mCherry* | Integrated, multicopy | Not tested | WY1331, WY1332, WY1333 | (5-10) |
| 8 | *wsp-1(crib)* | *sajIs31* | *mCherry* | Integrated, multicopy | Not tested | WY1374, WY1381, WY1382, WY1385 | (9) |
| 9 | *wsp-1(gbd)* | *xnIs506* | *gfp* | Integrated | Not tested | FT1459 | (11) |

1. Neukomm LJ, Zeng S, Frei AP, Huegli PA, Hengartner MO. Small GTPase CDC-42 promotes apoptotic cell corpse clearance in response to PAT-2 and CED-1 in C. elegans. Cell Death Differ. 2014;21(6):845-53.

2. Rodriguez J, Peglion F, Martin J, Hubatsch L, Reich J, Hirani N, et al. aPKC Cycles between Functionally Distinct PAR Protein Assemblies to Drive Cell Polarity. Dev Cell. 2017;42(4):400-15 e9.

3. Lazetic V, Fay DS. Conserved Ankyrin Repeat Proteins and Their NIMA Kinase Partners Regulate Extracellular Matrix Remodeling and Intracellular Trafficking in Caenorhabditis elegans. Genetics. 2017;205(1):273-93.

4. Yochem J, Lazetic V, Bell L, Chen L, Fay D. C. elegans NIMA-related kinases NEKL-2 and NEKL-3 are required for the completion of molting. Dev Biol. 2015;398(2):255-66.

5. Martin E, Harel S, Nkengfac B, Hamiche K, Neault M, Jenna S. pix-1 controls early elongation in parallel with mel-11 and let-502 in Caenorhabditis elegans. PLoS One. 2014;9(4):e94684.

6. Fotopoulos N, Wernike D, Chen Y, Makil N, Marte A, Piekny A. Caenorhabditis elegans anillin (ani-1) regulates neuroblast cytokinesis and epidermal morphogenesis during embryonic development. Dev Biol. 2013;383(1):61-74.

7. Martin E, Ouellette MH, Jenna S. Rac1/RhoA antagonism defines cell-to-cell heterogeneity during epidermal morphogenesis in nematodes. J Cell Biol. 2016;215(4):483-98.

8. Zaidel-Bar R, Joyce MJ, Lynch AM, Witte K, Audhya A, Hardin J. The F-BAR domain of SRGP-1 facilitates cell-cell adhesion during C. elegans morphogenesis. J Cell Biol. 2010;191(4):761-9.

9. Ouellette MH, Martin E, Lacoste-Caron G, Hamiche K, Jenna S. Spatial control of active CDC-42 during collective migration of hypodermal cells in Caenorhabditis elegans. J Mol Cell Biol. 2016;8(4):313-27.

10. Tsur A, Bening Abu-Shach U, Broday L. ULP-2 SUMO Protease Regulates E-Cadherin Recruitment to Adherens Junctions. Dev Cell. 2015;35(1):63-77.

11. Zilberman Y, Abrams J, Anderson DC, Nance J. Cdc42 regulates junctional actin but not cell polarization in the Caenorhabditis elegans epidermis. J Cell Biol. 2017;216(11):3729-44.
