# Supplementary material for: Actin organization and endocytic trafficking are controlled by a network linking NIMA-related kinases to the CDC-42-SID-3/ACK1 pathway
Source: PLoS Genet. 2018 Apr 2;14(4):e1007313. doi: 10.1371/journal.pgen.1007313 (PMC5897031; doi:10.1371/journal.pgen.1007313)
Supplement: S2 Table — Alignments of wild-type sequence and corresponding mutated regions in sid-3(fd213), sid-3(fd214), sid-3(fd218), sid-3(fd219), and sid-3(fd221) mutants. Mutated regions are flanked upstream and downstream with ten wild-type nucleotides. Deleted regions are marked in red; insertions are indicated in blue. The number of the first nucleotide in each sequence, based on sid-3 isoform a, is shown in parentheses. (DOCX) [file pgen.1007313.s013.docx]

| **Alleles** | **Modified regions** |
| --- | --- |
| Wild type | (5415) AGCCGATTCT CT C G TC T GAGGTGCTCC |
| *fd213* | (5415) AGCCGATTCT**ACAT**CT**AC**C**T**G**AA**TC**AGAGAT**T**C**GAGGTGCTCC |
| Wild type | (5412) CTGAGCCGAT**TCTCTCGTCTGAGG**TGCTCCAACC |
| *fd214* | (5412) CTGAGCCGAT TGCTCCAACC |
| Wild type | (47) TGCTCCGCAA**AGCAC**AGTTGGACGC |
| *fd218* | (47) TGCTCCGCAA AGTTGGACGC |
| Wild type | (52) CGCAAAGCAC**A**GTTGGACGCA |
| *fd219* | (52) CGCAAAGCAC GTTGGACGCA |
| Wild type | (51) CCGCAAAGCA **C**AGTTGGACGC |
| *fd221* | (51) CCGCAAAGCA**AGTAGCAAGTAGAAAGTAGCAAAGCAAAGCA** AGTTGGACGC |

**S1 Table. Mutated regions of CRISPR/Cas9-generated *sid-3* lines.** Alignments of wild-type sequence and corresponding mutated regions in *sid-3(fd213)*, *sid-3(fd214)*, *sid-3(fd218)*, *sid-3(fd219)*, and *sid-3(fd221)* mutants. Mutated regions are flanked upstream and downstream with ten wild-type nucleotides. Deleted regions are marked in red; insertions are indicated in blue. The number of the first nucleotide in each sequence, based on *sid-3* isoform a, is shown in parentheses.
